# Extracellular vesicles in cardiovascular diseases: pathogenic mediators, diagnostic tools, and therapeutic vectors

**DOI:** 10.3389/fcvm.2025.1666589

**Published:** 2025-10-08

**Authors:** Tianyi Li, Wan Wang, Zilu Qin, Yiling Chen, Kangjie Zhu, Haoyu Liu, Jiangjiang Sun, Hongpeng Zhong

**Affiliations:** ^1^ Xuzhou Key Laboratory of Laboratory Diagnostics, School of Medical Technology, Xuzhou Medical University, Xuzhou, China; ^2^ Xuzhou Key Laboratory of Laboratory Diagnostics, School of Anesthesiology, Xuzhou Medical University, Xuzhou, China

**Keywords:** extracellular vesicles, cardiovascular diseases, atherosclerosis, myocardial infarction, stroke, heart failure, hypertension, valvular heart disease

## Abstract

Cardiovascular diseases (CVDs), the leading global cause of mortality, underscore an urgent need for innovative diagnostic and therapeutic strategies. Extracellular vesicles (EVs)—lipid-bilayer nanoparticles transporting bioactive cargo (microRNAs, proteins, lipids)—are critical mediators of intercellular communication in CVD pathogenesis. They exhibit functional duality: propagating pathology (inflammation, fibrosis, thrombosis) while facilitating tissue repair. This review synthesizes EV biogenesis mechanisms, isolation methodologies, source-specific functions, and multifaceted roles in atherosclerosis, myocardial infarction, heart failure, and stroke. We further evaluate EV-based diagnostic biomarkers, engineered therapeutic applications, clinical translation challenges, and future directions.

## Graphical Abstract

**Figure:**
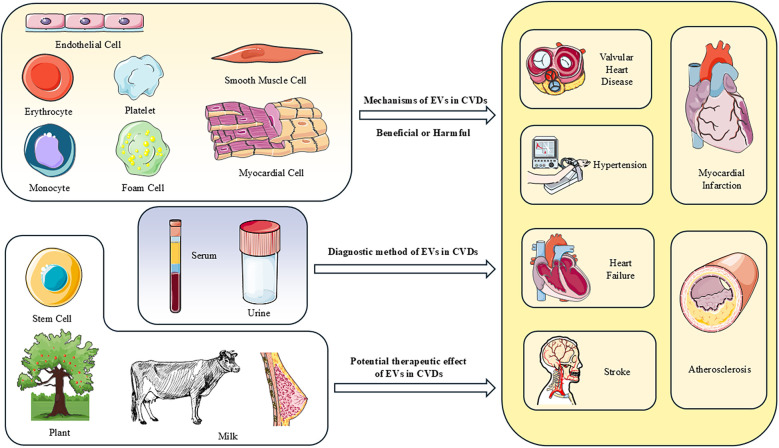
The roles of EVs in CVDs and the potential of acellular therapy. EVs derived from tissues and circulating sources target recipient cells through intercellular communication, influencing their biological functions and thereby impacting the progression of cardiovascular diseases. EVs from plant cells and stem cells carry molecules that promote protection and repair of the heart, significantly alleviating damage caused by cardiovascular diseases, indicating great potential as acellular therapies. EVs from plasma, serum and urine provide a potential avenue for the diagnosis and prognosis of future CVDs.

## 1 Introduction

In recent years, with the widespread spread of unhealthy living habits and the aggravation of the global aging trend, the morbidity and mortality of cardiovascular diseases (CVDs) have increased significantly, now accounting for approximately one-third of all deaths globally (1). CVDs encompass a diverse range of conditions affecting the heart and vascular system, categorized into several primary groups. Atherosclerosis and hypertension are prevalent conditions often regarded as major risk factors for various cardiovascular diseases, leading to complications such as myocardial infarction, heart failure, valvular heart disease, and stroke. Ischemic heart disease, characterized by insufficient blood supply to the heart, mostly refers to coronary heart disease (CHD), which can manifest as stable or unstable angina and myocardial infarction. Valvular heart disease involves damage to or a defect in one of the four heart valves, affecting blood flow through the heart. Finally, stroke, caused by interrupted blood supply or vessel rupture in the brain, encompasses ischemic and hemorrhagic types. This article primarily focuses on current research related to extracellular vesicles and their association with CVDs, as well as the closely related conditions in their pathogenesis. Despite significant advances in cardiovascular disease research, there remains an urgent need for the development of more effective preventive strategies, diagnostic tools, and therapeutic interventions. Extracellular vesicles (EVs) exhibit a dualistic nature in cardiovascular diseases (CVDs), functioning as both essential mediators of physiological homeostasis and potential instigators of pathological progression. Under physiological conditions, EVs serve as critical vectors for intercellular communication within the cardiovascular system. By selectively transferring bioactive molecules-including proteins, miRNAs, and lipids-between cells, EVs orchestrate vital processes such as angiogenesis, metabolic coordination, and tissue regeneration. This molecular crosstalk maintains vascular integrity, supports cardiac repair mechanisms, and ensures functional synergy among diverse cell types. Conversely, pathological contexts trigger EVs to adopt detrimental roles. Stress-induced alterations in EV biogenesis lead to the packaging of pro-inflammatory, pro-fibrotic, and pro-arrhythmic cargoes. These aberrant EVs propagate cellular damage by facilitating endothelial dysfunction, amplifying inflammatory cascades, and accelerating fibrotic remodeling. Moreover, they disrupt electrophysiological stability and promote maladaptive signaling across cardiac tissues. Systemically, EVs derived from injured cells disseminate pathological molecules to distant organs, exacerbating multi-organ crosstalk in conditions like heart failure. Furthermore, EVs demonstrate significant diagnostic utility (2). Circulating EVs encapsulate cell-specific molecular signatures shielded from degradation, offering a dynamic “liquid biopsy” platform (3). Quantitative and qualitative profiling of EV cargoes enables early detection of subclinical cardiovascular injury, stratifies disease severity, and predicts risks of major adverse events. Integration of multi-omics EV data with advanced analytics heralds a new era in precision cardiology, transforming EVs from biological messengers into powerful clinical tools. Given the breadth of cardiovascular phenotypes and the uneven volume of EV-specific evidence, this review focuses on atherosclerosis, myocardial infarction, heart failure, hypertension, and valvular heart disease; findings related to cardiomyopathies are discussed within the Heart Failure section where the clinical and pathophysiological features converge.

## 2 Overview of extracellular vesicles

### 2.1 Classification and biogenesis process of EVs

**Figure 1 F1:**
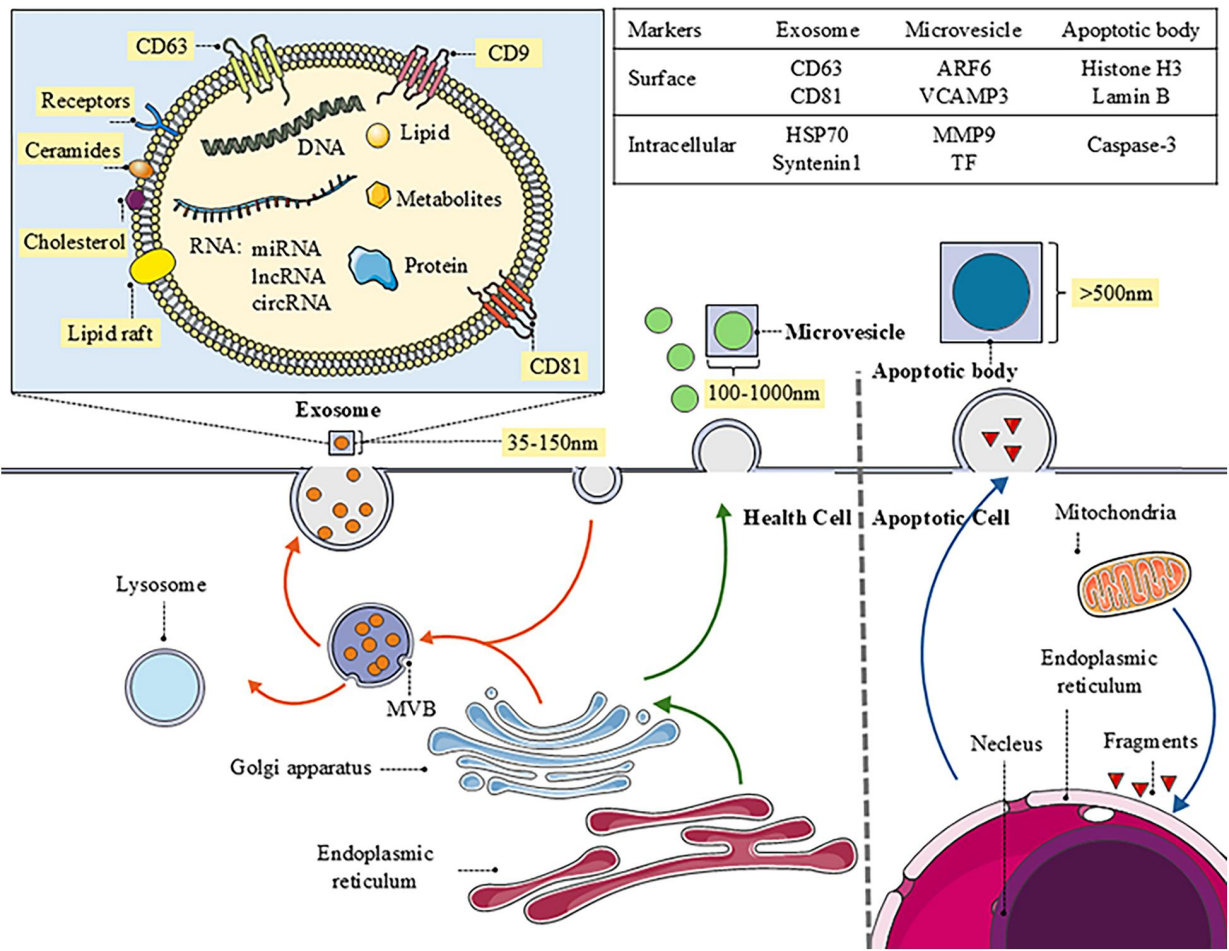
The biogenesis of EVs and the distinct characteristics of different types. The image provides a detailed depiction of the biogenesis and intricate molecular structure of exosomes, while briefly outlining the generation of microvesicles and apoptotic bodies. Additionally, it specifies the functions and markers associated with each type of vesicle, offering important insights into intercellular communication and their roles in biological processes.

Extracellular vesicles mainly include microvesicles, exosomes, and apoptotic bodies. They exhibit significant differences in size, composition, and surface markers (4) (Figure 1). Furthermore, the EVs research landscape is expanding to include other nanoscale vesicular structures, such as nanovesicles, which are typically defined by their small size (<50–100 nm) and are of emergent research interest due to their potential as efficient delivery vehicles (5). The article mainly talks about microvesicles and exosomes.[Fig F1]

Microvesicle (MV) biogenesis originates from the outward budding and fission of the plasma membrane. This process typically bypasses endosome formation and necessitates significant molecular reorganization within the plasma membrane, involving alterations in lipid composition, protein distribution, and calcium ion (Ca^2+^) concentration. Key facilitators, such as aminophospholipid translocases (flippases/floppases), scramblases, and calpain proteases, disrupt membrane phospholipid asymmetry. This disruption promotes physical membrane curvature, remodels the actin cytoskeleton, and ultimately enables membrane budding and MV release (6). The involvement of cytoskeletal components and their regulatory machinery is essential for MV generation (7). Accumulating evidence indicates a strong association between MV formation and the modulation of cytoskeletal dynamics by small GTPases, notably the Rho (RAS homology) family and ADP-ribosylation factor (ARF). In malignant cells, ROCK and ARF6 GTPases critically mediate vesicle budding by regulating cytoskeletal dynamics.

The generation of exosomes mainly involves five steps: endocytosis and inward budding of the plasma membrane, formation of early sorting endosomes (ESE), maturation of late sorting endosomes (LSE), formation of multivesicular bodies (MVB), and finally, the release of exosomes. First, exosome formation begins with the endocytosis of the cell membrane, allowing external substances to enter the cell and form ESEs, which can exchange substances with the trans-Golgi network and endoplasmic reticulum. During this process, the inward-budded membrane fuses with the endoplasmic reticulum, Golgi apparatus, and even mitochondria. ESEs mature into LSEs, where secondary inward budding results in the formation of intraluminal vesicles (ILV), which contain cellular components such as proteins, nucleic acids, and lipids (8). Thus, the formation of ILVs is one of the core steps in exosome biogenesis and is dependent on the endosomal sorting complex required for transport (ESCRT) complexes (ESCRT-0, -I, -II, and -III) and their core proteins, such as ESCRT-III, ALIX, and tumor susceptibility gene 101 (TSG101) (9). These proteins promote ILV generation by regulating inward budding of membranes and separation of vesicles (10). Subsequently, mature MVBs can either interact with intracellular autophagosomes or lysosomes for degradation or approach the cell membrane through the cellular cytoskeleton and microtubule system. When fused to lysosomes, ILVs are degraded, conversely, when they fuse with cell membranes, endosomes release exosomes into the extracellular space (11). In addition to the classical ESCRT-mediated pathway, exosome formation can also occur through ESCRT-independent pathways, such as those involving lipids and Rab GTPases. lipid raft-rich regions of the cell membrane may directly bend inward during endocytosis to form ILVs, a process that does not rely on ESCRT complexes but still requires specific lipids and membrane proteins, such as tetraspanins (e.g., CD63, CD81, etc.), which play essential roles in the formation and function of exosomes (12, 13). Similarly, Rab GTPases are involved in ESCRT-mediated exosome biogenesis and also regulate exosome formation independently of the ESCRT pathway. Rab GTPases, such as Rab27a, Rab35, and Rab11, promote the release of exosomes by regulating the trafficking of MVBs and their fusion with cell membranes.

Apoptotic bodies represent another category of EVs, generated through plasma membrane budding during programmed cell death (14). These vesicles are primarily cleared by phagocytes via efferocytosis. Regulation of apoptotic body formation involves specific molecular mediators governing apoptotic cell disassembly, including ROCK1 kinase, Pannexin 1 channels, and Plexin B2 receptors. Compared to exosomes and microvesicles, studies on apoptotic bodies as an EV subtype are limited (15).

From a functional and application perspective, EVs can be categorized into natural and artificial/synthetic classes. Natural EVs (like the aforementioned MVs and EXOs) carry bioactive molecules from their parent cells and act as crucial intercellular messengers, playing a dual role in the pathophysiology of cardiovascular diseases. In contrast, synthetic nanovesicles, including biomimetic vesicles based on cell membranes (e.g., platelet membrane, stem cell membrane vesicles) and synthetic liposomes, are primarily engineered to harness their potential as therapeutic delivery vehicles. By mimicking the membrane properties and targeting capabilities of natural EVs while allowing for precise engineering, these artificial nanovesicles can be efficiently loaded with drugs, nucleic acids (e.g., miRNA, siRNA), or functional nanoparticles for targeted delivery, overcoming the limitations of conventional therapies.

Evidence for their therapeutic potential is rapidly accumulating. Biomimetic nanovesicles designed for ischemic stroke therapy exemplify this promise. One innovative platform co-encapsulates thrombolytic tissue plasminogen activator (tPA) with neuroprotective melanin nanoparticles within a platelet membrane shell (16). This design leverages natural thrombus-targeting abilities for site-specific accumulation, followed by near-infrared light-triggered release of both cargoes. This approach not only accelerates clot lysis through thermal enhancement of tPA activity but also sequentially addresses ischemia-reperfusion injury through scavenging of reactive oxygen species, demonstrating sophisticated spatiotemporal control. Similarly, in atherosclerosis, engineered mesenchymal stem cell-derived nanovesicles have been functionalized with a novel targeting peptide (GSPREYTSYMPH, or PREY) selected through phage display screening (17). These PREY-conjugated nanovesicles exhibit precise homing to disturbed flow sites—early atherosclerotic lesions characterized by endothelial activation. Upon accumulation, they deliver their innate anti-inflammatory and pro-reparative cargo, effectively reducing endothelial permeability and monocyte recruitment in preclinical models. This strategy highlights how synthetic nanovesicles can be engineered to target specific cardiovascular pathophysiological niches for localized therapeutic intervention.

These emerging studies underscore the significant potential of nanovesicles, particularly through engineered designs, as next-generation precision platforms for cardiovascular drug delivery, offering solutions to longstanding challenges in targeting, controlled release, and combination therapy.

### 2.2 Isolation and separation of EVs

The isolation and enrichment of EVs constitute a critical prerequisite for their clinical application as diagnostic biomarkers and therapeutic agents (Table 1). Current mainstream separation techniques include ultracentrifugation (UC), density gradient ultracentrifugation, size-exclusion chromatography (SEC), ultrafiltration (UF), polymer-based precipitation, field-flow fractionation, and immunoaffinity capture (IA). Studies have demonstrated that polymer-based precipitation and size-exclusion chromatography yield the highest total EV production (18). Notably, asymmetric flow field-flow fractionation (AsFlFFF/AF4) has emerged as a promising technique for isolating EVs subpopulations (19). Microfluidic platforms and combinatorial multi-step approaches also exhibit unique potential in this field. Given the inherent biological complexity, source heterogeneity, and functional diversity of EVs, integrated separation strategies are generally considered optimal (20). Current technical challenges primarily involve exogenous contamination, laborious operational procedures, and standardization deficiencies, necessitating the development of novel separation-enrichment systems with enhanced purity and efficiency. For the distinct sources discussed herein, common EV isolation methods are as follows, Serum/plasma commonly employs UC or SEC (21). Urine frequently uses UF combined with SEC or polymer-based precipitation (22). Tissue-derived EVs require initial enzymatic digestion/homogenization, followed predominantly by density gradient ultracentrifugation. Stem cell supernatant often relies on UC, polymer-based precipitation, or SEC. Milk typically favors SEC or optimized density gradient ultracentrifugation (23). Plant-derived EVs mainly depend on differential centrifugation combined with density gradient centrifugation (24). Combinatorial approaches (e.g., UC + SEC) are widely adopted to enhance purity and yield for specific sources, and emerging technologies like microfluidics show promise for advancing EV isolation (25). Optimizing source-specific isolation strategies remains a key research priority.[Table T1]

**Table 1 T1:** Isolation methods of EVs (22, 26–34).

Methods	Abbreviation	Advantages	Disadvantages	Reference title
Differential/Ultracentrifugation	DC/UC	Cost-effective for large volumes	Long processing time	Improved recovery of urinary small extracellular vesicles by differential ultracentrifugation
		Simple protocols	Risk of EVs damage/aggregation	
		Accessible equipment	Protein co-isolation	
Density Gradient Centrifugation	DGC	High purity	Very time-consuming	Isolation of human salivary extracellular vesicles by iodixanol density gradient ultracentrifugation and their characterizations
		Preserves integrity	Low throughput; specialized equipment	
		Reduces aggregation	Risk of sample loss	
Size-Exclusion Chromatography	SEC	Rapid processing	Limited sample volume	A review of exosomal isolation methods: Is size exclusion chromatography the best option?
		High EVs/protein ratio	EVs dilution	
		Preserves function	Co-elution of contaminants	
Ultrafiltration/Tangential Flow Filtration	TFF	Rapid processing	Risk of membrane clogging	Extracellular vesicle isolation by a tangential-flow filtration-based large-scale purification method
		Reduced clogging	Shear stress lead to EVs deformation	
		Preserves integrity	Yield loss	
Polymer-Based Precipitation	PBP	Simple protocols	Co-precipitation of proteins or polymers	Enrichment of astrocyte-derived extracellular vesicles from human plasma
		High concentration yield	Contaminant interference in downstream analysis	
Immunoaffinity Capture	–	High specificity and purity	Low yield	EV separation: release of intact extracellular vesicles immunocaptured on magnetic particles
		subpopulation isolation	Expensive cost of antibodies	
		Works with small volumes	Excludes antigen-negative Evs	
Microfluidic-Based Isolation	–	Minimal sample use	Specialized fabrication	Recent advances in microfluidic-based extracellular vesicle analysis
		Automatable/high-throughput	Limited scalability	
Field-Flow Fractionation	FFF	High integrity	Specialized instrument	Separation and characterization of extracellular vesicles from human plasma by asymmetrical flow field-flow fractionation
		Isolates diverse EV subsets	Require pre-concentration	
Hydrostatic Dialysis Isolation	HDI	Effective abundant protein removal	Requires validation	MiR-145 detection in urinary extracellular vesicles increase diagnostic efficiency of prostate cancer based on hydrostatic filtration dialysis method
			Limited adoption	
Aqueous Two-Phase System	ATPS	Pure and scalable	Higher cost and longer time	Next generation aqueous two-phase system for gentle, effective, and timely extracellular vesicle isolation and transcriptomic analysis
		Gentle treatment	Limited clinical utility	

### 2.3 Different origins and functions of EVs

#### 2.3.1 EVs derived from plasma and serum

Mammalian blood is a primary source of extracellular vesicles (EVs), which can be isolated from either plasma or serum. Plasma is obtained from anticoagulated blood and retains coagulation factors, providing a representation of circulating EVs that is closer to the *in vivo* physiological state. In contrast, serum is derived from clotted blood, a process that potently activates platelets, leading to a significant ex vivo release of platelet-derived EVs and concomitant consumption of coagulation factors (35). Due to minimized ex vivo cellular activation, plasma-derived EVs are generally considered more reliable for reflecting the native circulating EV profile, exhibiting less pre-analytical variability and thus offering greater result reproducibility. Consequently, plasma is the preferred and most common source for EV research in cardiovascular diseases. However, serum serves a specific purpose in studies focusing on thrombosis. The very process of clot formation enriches serum with EVs released from platelets under low-flow and hypercoagulable conditions ex vivo, making it a valuable medium for investigating the role of platelet-derived EVs in thrombotic pathologies (36). Both plasma-EVs and serum-EVs originate from various cellular sources and serve as critical mediators of intercellular communication and clinically validated diagnostic biomarkers. They encapsulate cell-state-specific cargoes (e.g., miRNAs, proteins) that reflect real-time pathophysiological states. For instance, sEV-derived miR-1 and miR-133a demonstrate significant elevation within 3 h post-infarction, serving as early-warning signatures for acute myocardial infarction (AMI) prior to troponin detectability (37). Their intrinsic biocompatibility and circulatory stability further underscore their potential for targeted therapeutic delivery.

#### 2.3.2 EVs derived from milk

Milk contains high amounts of EVs originating from different cell populations in the mammary glands (e.g., adipocytes, epithelial cells, stem cells, and immune cells) and plays important roles in interorganismal and cross-species communication. Because milk is considered scalable, economical, and safe source of EVs, milk-derived EVs (miEVs) have recently been introduced as novel drug carriers (38). A significant advantage of miEVs is their suitability for oral administration, which is primarily attributed to their innate resistance to degradation in the harsh conditions of the gastrointestinal tract (39). Milk contains a high concentration of casein micelles, and these phosphoproteins exhibit chaperone-like properties that can encapsulate and protect EVs during transit through acidic environments. This protective effect facilitates the efficient uptake of EVs by intestinal epithelial cells, thereby enabling the systemic distribution of their bioactive cargos. Studies using cardiosphere-derived cells EVs (CDC-EVs) have illustrated that the association with casein enhances EV uptake and biodistribution, leading to improved tissue targeting and therapeutic outcomes in animal models (40).

#### 2.3.3 Evs derived from plant

The Plant-Derived Extracellular Vesicles (PDEVs) are nano-sized vesicle structures ranging from 50 to 1,000 nm in diameter, featuring a spherical lipid bilayer. Importantly, the human immune system does not detect these PDEVs, allowing them to achieve longer circulation times and higher bioavailability (41). Furthermore, PDEVs exhibit the ability to penetrate the intestinal mucus barrier and withstand gastrointestinal enzymes and bile, making them a reliable and advantageous choice for drug delivery. They carry small RNAs and miRNAs that facilitate intercellular communication among species across different kingdoms. As natural vectors, PDEVs transport bioactive compounds derived from their source plants. In addition to their functional roles, PDEVs demonstrate significant biological activities, such as antioxidant and anti-inflammatory effects (42). Notably, their outer lipid bilayer membrane acts as a protective barrier for bioactive molecules against external conditions. This structural feature not only allows PDEVs to sustain circulation within the body for extended periods but also promotes the continuous accumulation of drugs. Moreover, PDEVs are known for their strong safety profile, exhibiting low toxicity and good biocompatibility while minimizing side effects, which further enhances their potential as effective drug delivery systems.

## 3 Mechanisms of EVs in CVDs

### 3.1 Mechanisms of EVs in atherosclerosis

#### 3.1.1 Endothelial dysfunction and vascular inflammation

Atherosclerosis is fundamentally a chronic inflammatory and fibroproliferative response to retained and modified lipoproteins. Classical risk factors reduce endothelial nitric oxide bioavailability and increase permeability and adhesion molecules, promoting monocyte recruitment and transendothelial migration (43). Within the intima, monocytes differentiate into macrophages and foam cells; activated platelets amplify leukocyte recruitment and coagulation, whereas smooth muscle cells migrate and produce matrix, driving plaque growth, calcification, and instability (44). Throughout these steps, EVs shuttle miRNAs, proteins, and lipids that fine-tune the endothelial–monocyte/macrophage–platelet–smooth muscle cross-talk: some EVs propagate oxidative stress, inflammation, and thrombosis, while others favor resolution and repair (45, 46). This framework underpins the mechanistic and translational studies summarized below (Tables 2, 3).[Table T2][Table T3]

**Table 2 T2:** Damage effects of EVs in AS (47–49, 54, 56–64).

Source	Cargos	Signaling pathway/target	Function	Reference title
ox-LDL-treated ECs	miR-505	SIRT3↓	Promotes NET formation → Aggravates endothelial inflammation	Exosome-encapsulated miR-505 from ox-LDL-treated vascular endothelial cells aggravates atherosclerosis by inducing NET formation
ox-LDL-treated ECs	HIF1A-AS2	miR-455-5p↓ → ESRRG↑ → NLRP3↑	Induces endothelial pyroptosis and vascular inflammation	Endothelial cell-released extracellular vesicles trigger pyroptosis and vascular inflammation to induce atherosclerosis
ox-LDL-treated ECs	LINC01005	miR-128-3p↓ → KLF4↑	Drives VSMC phenotypic switching → Endothelial injury	Exosomal LINC01005 derived from oxidized low-density lipoprotein-treated endothelial cells regulates vascular smooth muscle cell phenotypic switch
High glucose-treated ECs	PDGF-BB	miR-296-5p↑ → Bcl-2↑Bak/Bax↓	Suppresses VSMC apoptosis → Abnormal angiogenesis	PDGF-BB carried by endothelial cell-derived extracellular vesicles reduces vascular smooth muscle cell apoptosis in diabetes
Obese visceral adipose tissue	miR-27b-3p	PPARα↓ → NF-κB↑	Induces endothelial inflammation	Exosomal miR-27b-3p secreted by visceral adipocytes contributes to endothelial inflammation and atherogenesis
High Glucose + ox-LDL treated Ecs	NEDD4l	IκB-α/PPAR*γ*↓	Promotes macrophage M1ɸ polarization and ox-LDL uptake	Exosomal NEDD4l derived from HG + oxLDL-induced vascular endothelial cells accelerates macrophage M1 polarization and oxLDL uptake by ubiquitinating IκBα and PPARγ.
High Glucose + AngII treated Ecs	–	eNOS↓and ERK↑	Reduces NO bioavailability → increases permeability	Glucose and angiotensin II-derived endothelial extracellular vesicles regulate endothelial dysfunction via ERK1/2 activation.
Radiation-treated Ecs	miR-126-5p/miR-212-3p	Monocytes↑	Triggers vascular inflammation	The miR-126-5p and miR-212-3p in the extracellular vesicles activate monocytes in the early stage of radiation-induced vascular inflammation implicated in atherosclerosis
Platelets	miR-92a-3p	PTEN/PIP3/Akt → Col8a1↑	Induces vascular stiffness and reduced elasticity	Platelet-Derived Extracellular Vesicles Increase Col8a1 Secretion and Vascular Stiffness in Intimal Injury
M1 macrophages	miR-185-3p	Smad7↓ → TGF-β↑	Inhibits EC proliferation → Promotes EC apoptosis	M1 macrophages-derived extracellular vesicles elevate microRNA-185-3p to aggravate atherosclerosis
ox-LDL-treated macrophages	miR-19b-3p	JAZF1↓	Enhances VSMC migration/proliferation	microRNA-19b-3p-containing extracellular vesicles derived from macrophages promote the development of atherosclerosis by targeting JAZF1
Mature dendritic cells	TNF-α	NF-κB↑	Induces endothelial injury	Exosomes derived from mature dendritic cells increase endothelial inflammation and atherosclerosis via membrane TNF-α mediated NF-κB pathway
Neutrophils	miR-155	BCL6↓	Exacerbates endothelial inflammation	Neutrophil microvesicles drive atherosclerosis by delivering miR-155 to atheroprone endothelium
Nicotine exposure (via MEVs)	miR-155	–	Worsens endothelial damage	Nicotine exacerbates endothelial dysfunction and drives atherosclerosis via extracellular vesicle-miRNA
NAFLD hepatocytes	miR-1	KLF4/NF-κB↑	Triggers endothelial inflammation	Hepatocyte-derived extracellular vesicles promote endothelial inflammation and atherogenesis via microRNA-1
Sleep deprivation	miR-182-5p↓	MYD88↓ → NF-ĸB/NLRP3↓	Promotes endothelial inflammation	Sleep deprivation promotes endothelial inflammation and atherogenesis by reducing exosomal miR-182-5p
TNF-α/shear stress-treated ECs	miR-92a	KLF4↓	Accelerates LDL uptake → Foam cell formation	Extracellular MicroRNA-92a mediates endothelial cell-macrophage communication
H. pylori Bacteria	Bacterial antigens	IL-6/IL-8↑	Mediates inflammation and endothelial injury	Extracellular vesicles and endothelial dysfunction in infectious diseases.

**Table 3 T3:** Repair effect of EVs in AS (65–74).

Source	Cargos	Signaling pathway/target	Function	Reference title
MSCs	miR-let7	HMGA2/IGF2BP1↓ → NF-κB/PTEN↓	Promotes M2 polarization → Anti-inflammation	Exosomes derived from mesenchymal stem cells attenuate the progression of atherosclerosis in ApoE(−/−) mice via miR-let7 mediated infiltration and polarization of M2 macrophage
MSCs	TGF-β1	miR-132↑ → Mycbp2/TSC2↑	Induces M2 polarization → Inhibits calcification	Mesenchymal stem cell-secreted extracellular vesicles carrying TGF-β1 up-regulate miR-132 and promote mouse M2 macrophage polarization
MSCs	miR-145	JAM-A↓	Inhibits EC migration → Reduces inflammation	Mesenchymal stem-cell-derived exosomal miR-145 inhibits atherosclerosis by targeting JAM-A
MSCs	FENDRR	miR-28↓ → TEAD1↑	Suppresses EC apoptosis/oxidative stress	Exosomes derived from mesenchymal stem cells ameliorate the progression of atherosclerosis in ApoE(−/) mice via FENDRR
Thrombin-activated platelets	miR-223	ICAM-1↓ → NF-κB/MAPK↓	Attenuates endothelial inflammation	Thrombin-activated platelet-derived exosomes regulate endothelial cell expression of ICAM-1 via microRNA-223
LSW-treated VSMCs	miR-145	PDCD4↓	Improves EC proliferation/migration	Tripeptide Leu-Ser-Trp regulates the vascular endothelial cells phenotype switching by mediating the vascular smooth muscle cells-derived small extracellular vesicles packaging
Endothelial cells	miR-222	ICAM-1↓	Inhibits monocyte adhesion to ECs	Endothelial microparticles reduce ICAM-1 expression in a microRNA-222-dependent mechanism
M2 macrophages	miR-221-3p	Grb10↓	Reduces EC apoptosis/inflammation	M2 macrophage-derived exosomes inhibit apoptosis of HUVEC cell through regulating miR-221-3p
Apoptotic ECs	miR-126	RGS16↓ → CXCR4/CXCL12↑	Recruits progenitor cells → Endothelial repair	Delivery of microRNA-126 by apoptotic bodies induces CXCL12-dependent vascular protection
Perivascular adipose tissues	miR-382-5p	BMP4↑ → PPARγ-ABCA1/ABCG1↑	Enhances cholesterol efflux → Reduces foam cells	Perivascular adipose-derived exosomes exert proatherogenic effects by regulating macrophage foam cell formation and polarization
Endothelial progenitor cells	miR-21-5p	SIPA1L2↓ → Autophagy↑	Promotes EC proliferation/migration → Vascular repair	Endothelial colony-forming cell-derived exosomes regulate autophagic flux to promote vascular endothelial repair
HAL-loaded M2 macrophages	HAL (drug)	Heme synthesis↑ → CO/bilirubin↑	Exerts anti-inflammatory effects	Molecularly engineered macrophage-derived exosomes with inflammation tropism and intrinsic heme biosynthesis for atherosclerosis treatment
Lemon-derived EVs	-	AhR/Nrf2↑	Antioxidant/anti-inflammatory effects	Lemon-derived nanovesicles achieve antioxidant and anti-inflammatory effects activating the AhR/Nrf2 signaling pathway
Paeonol-treated monocytes	miR-223	STAT3↓	Reduces EC adhesion/inflammation	Paeonol attenuated inflammatory response of endothelial cells via stimulating monocytes-derived exosomal MicroRNA-223
Genetically modified ECs	anti-miR-33a-5p	miR-33a-5p↓ → ABCA1↑	Enhances cholesterol efflux	Exosome-mediated transfer of anti-miR-33a-5p from transduced endothelial cells enhances macrophage and vascular smooth muscle cell cholesterol efflux
Foam Cells	Prosaposin	GPR37↓ → ERK/AP1↑ → Tim4↑	Enhances efferocytosis and accelerates inflammation resolution	Efferocytes release extracellular vesicles to resolve inflammation and tissue injury via prosaposin-GPR37 signaling
Avocado-derived EVs	ginkgetin and berberine	TNF-α/IL-6/IL-1β/CD36↓	Inhibits oxLDL-induced foam cell formation	Avocado-derived extracellular vesicles loaded with ginkgetin and berberine prevent inflammation and macrophage foam cell formation.
Quercetin-loaded EVs	Quercetin	SIRT1↑	Alleviates ferroptosis and aging-related endothelial damage	Therapeutic application of quercetin in aging-related diseases: SIRT1 as a potential mechanism.

##### 3.1.1.1 Endothelial cell-derived EVs in AS

The inducing factors for EVs that cause endothelial cell injury partially to overlap with traditional cardiovascular disease (CVD) risk factors, including but not limited to: diabetes, hyperlipidemia, hypertension, and smoking. The exosomal cargo NEDD4l, released by vascular endothelial cells under high glucose and ox-LDL induction, enhances the ubiquitination of IκB-α and PPAR*γ*, promotes macrophage M1 polarization and ox-LDL uptake, thereby exacerbating endothelial injury (47). Under high glucose and AngII stimulation, endothelial cell-derived EVs reduce NO bioavailability by inhibiting endothelial nitric oxide synthase (eNOS) activity and activating the ERK signaling pathway, thereby promoting endothelial dysfunction and increasing permeability (48). Endothelial cell-derived EVs containing miR-126-5p and miR-212-3p are released after radiation exposure, triggering vascular inflammation via monocyte activation (49) This early dysfunction facilitates leukocyte adhesion and trans-endothelial migration, initiating the inflammatory cascade in the arterial intima.

##### 3.1.1.2 Monocyte cell-derived EVs in AS

During the development of atherosclerosis, following endothelial dysfunction, the next significant contributor to AS is foam cell formation. Under the influence of various adhesion and chemotactic factors, monocytes adhere to the damaged vascular endothelium, migrate across the vascular wall, and transform into macrophages. Macrophages can uptake erythrocyte-derived extracellular vesicles via endocytosis (50). Under inflammatory stimulation, their cargo heme can attenuate the ability of ox-LDL-treated macrophages to form foam cells. Foam cell-derived EVs express prosaposin, which binds to macrophage GRP37, increasing the expression of the efferocytosis receptor Tim4 through activation of the ERK-AP1 signaling axis (51). This leads to enhanced macrophage efferocytosis efficiency and accelerated inflammation resolution. Exosomes (BMDM-IL-4-exo) produced by bone marrow-derived macrophages (BMDMs) exposed to the M2-polarizing cytokine IL-4 contain abundant miR-99a/146b/378a (52). These exosomal miRNAs inhibit inflammation by targeting NF-κB and TNF-α signaling and further promote M2 polarization in recipient macrophages. Under high glucose induction, extracellular vesicles released by bone marrow cell-derived macrophages increase the number of circulating hematopoietic and myeloid cells. This leads to an increased macrophage population, exacerbating vascular inflammation and promoting atherosclerosis progression. Under nicotine stimulation, EVs derived from monocytes are secreted abundantly. These EVs encapsulate miR-155, which induces endothelial cell dysfunction and vascular inflammation by targeting BCL2, MCL1, TIMP3, BCL6 and activating the NF-κB pathway (53). During atherosclerosis progression, foam cell formation represents a pivotal pathological stage following endothelial dysfunction, wherein macrophages differentiated from migrated monocytes regulate inflammation and foam cell dynamics via EVs uptake; EVs derived from diverse cellular sources and their miRNA cargo exert bidirectional modulation, either resolving inflammation or exacerbating vascular damage through targeted signaling cascades, collectively driving disease advancement.

##### 3.1.1.3 Platelet-derived EVs in AS

Platelet-derived EVs modulate vascular homeostasis through targeted miRNA delivery, exerting dual regulatory effects that suppress endothelial inflammation under physiological conditions while promoting vascular stiffening and prothrombotic states in pathological contexts. Platelet-secreted EVs participate in delivering miR-92a-3p to vascular smooth muscle cells (VSMCs) (54). This then induces the production and secretion of Col8a1 via the PTEN/PIP3/Akt pathway, leading to increased vascular wall stiffness and reduced elasticity (54). Thrombin-activated platelet-derived exosomes (P-EXO) release large amounts of miR-223, which subsequently inhibits ICAM-1 expression in endothelial cells (ECs). MiR-223 may prevent EC inflammation by modulating NF-kB and MAPK pathways (55). Simultaneously, they transport high levels of miR-25-3p, which is responsible for reducing ox-LDL-induced EC inflammation and lipid deposition, thereby inhibiting AS progression. Platelet-derived exosomes containing miRNAs such as miR-223 and miR-25-3p can, under certain conditions, modulate endothelial activation and reduce inflammation, however, in pro-inflammatory states, these EVs may also promote a procoagulant environment by delivering tissue factor (TF) and plasminogen activator inhibitor-1 (PAI-1).

##### 3.1.1.4 Other sources EVs in AS

Steatotic hepatocyte-derived EVs promote endothelial inflammation by mediating miR-1-induced KLF4 pathway suppression and NF-κB pathway activation (75). Human aortic stenotic valve-derived extracellular vesicles can lead to endothelial dysfunction, pro-adhesive and procoagulant responses via the AT1R/NADPH oxidase/sodium-glucose cotransporter 2 (SGLT2) pro-oxidative pathway (76). Extracellular vesicles derived from human induced pluripotent stem cell-derived endothelial cells (hiPSC-ECs) reduce endothelial cell apoptosis and restore autophagy, suggesting that EV-based therapies offer a promising avenue for targeting endothelial dysfunction in similar vascular pathologies (77). During Helicobacter pylori infection, bacterium-derived EVs deliver bacterial antigens into vascular endothelial cells, mediating inflammation and endothelial injury (64). Notably, milk-derived EVs enriched with miR-30b-5p have been implicated in cardiovascular and metabolic diseases by modulating inflammation and oxidative stress. This miRNA suppresses TLR4 activity in endothelial cells, downregulates pro-inflammatory molecules, and thereby attenuates atherosclerotic progression (78). In atherosclerosis, EVs derived from heterogeneous cellular sources can pathologically exacerbate endothelial damage and procoagulant responses, while therapeutically attenuating apoptosis and restoring autophagic homeostasis.

##### 3.1.1.5 Potential therapeutic effect of PDEVs in AS

Many studies indicate that plant-derived extracellular vesicles (PDEV) can deliver antioxidants as a therapeutic application for oxidative stress-related diseases. MiRNAs in green leafy vegetable-derived EVs (such as miR-156a) have been shown to exert protective effects against atherosclerosis by modulating gene expression in human cells. Furthermore, plant compounds with antioxidant activity known to be carried in these vesicles (e.g., polyphenols and flavonoids) can scavenge reactive oxygen species and upregulate protective pathways like Nrf2 (42). These effects may reduce endothelial oxidative stress and the production of inflammatory mediators, both key to the pathogenesis of vascular inflammation. In a study investigating avocado-derived extracellular vesicles, researchers demonstrated a significant reduction in macrophage inflammatory cytokine expression (including TNF-α, IL-6, IL-1β, and Cd36) and inhibition of oxLDL-induced foam cell formation (79). EVs serve as vehicles for quercetin loading and delivery, mediating SIRT1 activation to alleviate ferroptosis and prevent aging-related diseases (80). This study highlights the potential of EVs as signaling carriers for treating aging-related diseases.

#### 3.1.2 Plaque formation, calcification & destabilization

Monocyte recruitment and foam cell accumulation secondary to endothelial dysfunction are hallmarks of atherosclerotic plaque growth. Following initial plaque formation and VSMC proliferation and migration, calcium deposition can begin, particularly within the fibrous cap covering the plaque. The calcification process plays a crucial role in plaque vulnerability.

Under endotoxin stimulation, monocyte-derived microvesicles can transmit cell death signals via encapsulated caspase-1, inducing programmed apoptosis in VSMCs, thereby promoting the calcification process and plaque formation (81). EV-derived circ_0001785 has been identified as a novel biomarker for atherosclerosis and has been demonstrated to reduce endothelial cell injury and delay plaque formation via the miR-513a-5p/TGFBR3 ceRNA network mechanism (82). Engineering extracellular vesicles offers a potential EV-based therapeutic strategy for atherosclerosis generation. Small extracellular vesicles (psEVs) derived from carotid artery plaques provide insights into tissue- and disease-specific pathology. PsEVs have been demonstrated to induce inflammatory endothelial dysfunction *in vitro* and exacerbate atherogenesis in ApoE-deficient mice (83). During endothelial cell apoptosis, CXCL12 production mediated by miR-126, and released via apoptotic bodies, promotes progenitor cell recruitment through paracrine action and inhibits macrophage adhesion to the vascular wall, thereby limiting atherosclerosis progression (72). Mouse experiments have demonstrated that administering CXCL12-containing apoptotic bodies or miR-126 confers plaque-stabilizing characteristics in different atherosclerotic mouse models.

As plaques progress, they undergo complex remodeling processes, including calcification and fibrous cap destabilization. EVs released by VSMCs and macrophages are implicated in promoting calcification and altering plaque stability. Calcifying EVs are a subset of EVs enriched in calcium-binding proteins (such as annexins) and can serve as nucleation factors for hydroxyapatite crystals, thereby promoting microcalcification within plaques. A biomimetic nanocarrier modeled after natural grapefruit-derived extracellular vesicles was designed and manufactured (84). It is loaded with sodium thiosulfate (STS, an approved drug for treating vascular calcification—VC) and further modified with an elastin-specific targeting peptide (ESTP) for VC-targeted delivery of STS. In *in vitro* experiments, ESTP nanomedicine demonstrated superior cellular uptake in calcifying vascular smooth muscle cells. Through the absorption of delivered STS, it inhibited VSMC calcification. Mechanistically, ESTP nanomedicine significantly prevented VC by driving M2 macrophage polarization, reducing inflammation, and inhibiting the bone-vascular axis.

Furthermore, EV-mediated delivery of matrix metalloproteinases (MMPs) and other proteolytic enzymes to the extracellular matrix can degrade collagen and other structural proteins within the fibrous cap. This degradation not only weakens the fibrous cap strength but also increases the risk of plaque rupture and subsequent thrombus formation. Plaque rupture is often the triggering event for acute thrombotic cardiovascular events. EVs promote the thrombotic cascade by providing a procoagulant surface rich in phosphatidylserine (PS) and tissue factor (TF). Specifically, platelet-derived EVs can enhance the coagulation process by transferring TF to the surfaces of endothelial cells and monocytes, thereby augmenting thrombin generation and local clot formation (85). EVs from monocyte/platelet aggregates can stimulate the release of proinflammatory cytokines from the plaque, further exacerbating the local prothrombotic microenvironment, secondary to other types of cardiovascular diseases (86). EVs exert dual regulatory effects both beneficial and detrimental on the inflammation-fibrosis-calcification axis during the development of atherosclerotic disease. On one hand, ox-LDL infiltrates endothelial cells, and various EVs released by endothelial cells recruit the mononuclear lymphatic system, promoting endothelial inflammation. This leads to subsequent endothelial fibrosis and eventual calcification detachment in the progression of atherosclerosis. On the other hand, EVs derived from endothelium and plaques suppress endothelial inflammation and plaque detachment, thus playing a beneficial role in disease progression.

### 3.2 Mechanisms of EVs in hypertension

#### 3.2.1 Mechanisms of EVs in hypertension damage

The pathogenic role of EVs in hypertension shares several key mechanisms with atherosclerosis, particularly involving chronic inflammation, oxidative stress, and dysregulated vascular tone. In hypertension, EVs derived from activated vascular endothelial cells, platelets, and immune cells propagate vascular dysfunction by transferring proinflammatory cytokines, signaling receptors, and regulatory RNAs to recipient cells (87). For instance, endothelial cell-derived EVs impair eNOS activity, thereby reducing nitric oxide (NO) bioavailability, a key factor in maintaining vascular tone. Moreover, EVs enriched in molecules such as TNF-α and IL-1β can activate NF-κB signaling in vascular smooth muscle and endothelial cells, promoting a proinflammatory environment that contributes to vascular remodeling and stiffness. EVs also contribute to the dysregulation of the renin-angiotensin system (RAS) by transporting components like the angiotensin II type 1 receptor (AT1R), which can be transferred to recipient cells to potentiate the local RAS response and facilitate processes like hypertrophy and remodeling (88). Furthermore, beyond the vasculature, brain-derived EVs have been shown to induce neuroinflammation and oxidative stress within key cardiovascular regulatory regions of the brain, such as the paraventricular nucleus (89). These EVs carry proinflammatory cytokines and enzymes that promote mitochondrial dysfunction and reactive oxygen species production, perpetuating a state of oxidative stress that increases sympathetic nervous system activity, a recognized driver of hypertension (90). The molecular cargo of EVs differs significantly between hypertensive and normotensive states, underscoring their active role in both perpetuating disease and potentially offering pathways for intervention.

#### 3.2.2 Diagnostic potential of EVs in hypertension

At present, the diagnosis and clinical management of essential hypertension continue to rely predominantly on conventional blood pressure (BP) measurements (91). While these measurements remain fundamental to hypertension assessment, they cannot differentiate between hypertensive subtypes, identify underlying molecular mechanisms, or optimally monitor treatment efficacy (92). This has created a compelling need for novel biomarkers that facilitate early detection, enable patient stratification, and allow monitoring of cardiovascular complications. EVs have recently emerged as promising candidate biomarkers in cardiovascular research due to their stability in circulation, ease of isolation from accessible bodily fluids, and, most importantly, their molecular cargo that dynamically reflects cardiovascular stress responses and pathological remodeling (93).

Evidence from multiple studies indicates that plasma-derived and urinary-derived EVs carry distinctive molecular signatures associated with hypertensive cardiovascular damage (94). Clinical studies have established that circulating platelet-derived EVs show significant correlations with nocturnal blood pressure patterns, non-dipping status, and increased pulse wave velocity, a validated marker of arterial stiffness, establishing these EVs as integrated biomarkers of vascular health status (95). Furthermore, research has indicated that endothelial-derived EVs carrying angiotensin II type 1 receptors (AT1R) exhibit significantly elevated levels in hypertensive patients and potentially modulate vascular responses to neurohormonal activation (96).

The miRNA content of EVs provides particularly valuable insights into hypertension-related cardiovascular pathology. Upregulated miRNAs such as miR-320d and miR-423-5p have been mechanistically implicated in promoting vascular smooth muscle phenotype switching and vascular remodeling (97). Furthermore, studies have identified that EVs from injured endothelial cells can remodel the vessel wall in hypertension through these miRNA-mediated mechanisms (98). Proteomic analyses have also revealed significant alterations in EV protein cargo; for instance, hypoxia-induced EVs carry glucose-regulated protein 78 kDa (GRP78), which contributes to vascular smooth muscle cell calcification—a process relevant to hypertension-mediated cardiovascular damage (99). Another study demonstrated that circulating EV levels were significantly different between white coat hypertension and sustained hypertension phenotypes, suggesting their utility in hypertension subtyping (95).

Emerging technologies are enhancing the diagnostic potential of EV-based biomarkers. Analysis of plasma small extracellular vesicles (sEVs) using surface-enhanced Raman scattering (SERS) combined with machine learning methods has demonstrated high sensitivity and specificity for cardiovascular disease detection (100). This interdisciplinary approach offers a promising strategy for non-invasive, precise early detection of hypertension-related cardiovascular impairment. Additionally, research into Mendelian hypertension forms has discovered EV-mediated alterations in vascular function, particularly through mechanisms involving vascular smooth muscle calcification and endothelial dysfunction (101).

The multifaceted information contained within EVs, reflecting various aspects of cardiovascular pathophysiology, offers a promising platform for improved risk stratification and dynamic monitoring of hypertensive patients beyond conventional BP assessment. Their ability to provide insights into vascular stiffness, myocardial stress, and vascular remodeling makes them particularly valuable for managing hypertension-related cardiovascular complications.

### 3.3 Mechanisms of EVs in myocardial infarction and ischemia/reperfusion injury

Over the past five years, while research on extracellular vesicles (EVs) in the AS (atherosclerosis) field has primarily focused on pathogenic mechanisms and cell-free therapies, the main research focus on EVs in myocardial ischemia, myocardial infarction (MI), and ischemia-reperfusion injury (I/R) has centered on stem cell therapy. The etiology of these three diseases is attributed to vascular occlusion, caused by various factors including atherosclerosis, thrombosis, and other pathological processes, ultimately leading to cardiac ischemia. Myocardial ischemia refers to the deficiency of oxygen and nutrients in the myocardium due to insufficient blood supply. If this state persists, it may progress to myocardial infarction, characterized by irreversible necrosis of cardiomyocytes. Furthermore, during reperfusion following myocardial infarction, the restoration of blood flow can induce ischemia-reperfusion injury, which further exacerbates cardiomyocyte damage and impacts patient prognosis. Therefore, understanding these pathophysiological processes is crucial for developing effective clinical interventions.

#### 3.3.1 Mechanisms of EVs in MI damage

Extracellular vesicles from CDC-EVs enhance regulatory T cell (Treg) proliferation and interleukin-10 (IL-10) production. BCYRN1, a long non-coding RNA (lncRNA) highly abundant in CDC-EVs, plays a significant role (102). In mouse models of myocardial infarction and reperfusion injury, administration of CDC-EVs, particularly those overexpressing BCYRN1, demonstrated cardioprotective effects by reducing infarct size and troponin I levels. M2 macrophage-derived extracellular vesicles containing miR-378a-3p in cardiomyocyte pyroptosis after MI disrupt NLRP3 and inhibit activation of the NLRP3/Caspase-1/GSDMD pathway by suppressing ELAVL1 (HuR) expression and HuR translocation to the cytoplasm, thereby alleviating cardiomyocyte pyroptosis (103). Recent studies indicate that EVs derived from coronary injury sites in MI patients, particularly leukocyte-derived EVs carrying malondialdehyde (MDA+) oxidation epitopes, activate neutrophils via the TLR4/PAD4 signaling pathway, inducing NETosis, thereby exacerbating local thrombosis and myocardial injury (104). Naturally occurring human MDA-specific IgM antibodies (MDA-IgM) significantly inhibit NETosis. A strong negative correlation was verified between the CD45 + MDA + EVs/IgM ratio and worsening cardiac function. Findings suggest that M2 macrophage-derived small extracellular vesicles (SEVs) containing circUbe3a promote proliferation, migration, and phenotypic transformation of cardiac fibroblasts (CFs) by directly targeting the miR-138-5p/RhoC axis, which may also exacerbate myocardial fibrosis after acute myocardial infarction (105). The EVs/macrophage axis plays a role in exacerbating I/R injury, similar to its function in damaging vascular endothelium in AS. EVs can influence the progression of myocardial ischemia-reperfusion injury by affecting macrophage polarization. miR-155-5p in heart-derived EVs promotes macrophage M1 polarization by activating the JAK2/STAT1 pathway, leading to local cardiac inflammation and even triggering systemic inflammation in distant organs (106). During MI progression, EVs orchestrate a paradoxical landscape of protective regeneration vs. pathological damage through spatiotemporally constrained immunomodulatory and cell death pathway regulation.

#### 3.3.2 Diagnostic effect of EVs in MI

Currently, the standard biomarkers for myocardial infarction (MI) are high-sensitivity cardiac troponins, including cTn-I and cTn-T (107). Although highly effective in the clinical setting for diagnosing MI, high-sensitivity troponins focus solely on AMI diagnosis and not on prediction and early warning. Therefore, finding new biomarkers is necessary, as early diagnosis of MI is associated with improved outcomes for future MI development. EVs have garnered considerable interest as novel blood biomarkers (Table 4). EVs have become important diagnostic tools for ischemic heart disease (IHD) patients because they can be easily enriched in large quantities from bodily fluids, and their diverse cargo is resistant to degradation (108). The cargo of EVs reflects the true metabolic state of the originating cardiomyocytes. Studies have indicated that after acute myocardial infarction (AMI), compared to the control group, EVs in the blood of the AMI group are larger in size, with elevated circulating levels of CD144 but decreased levels of CCR6 and CXCR3 (109). Simultaneously, compared to peripheral blood after AMI, coronary artery blood showed a significant decrease in CCR6 levels. Multiple experiments have demonstrated that a hypoxic environment promotes cardiomyocyte secretion of EVs. Using surface antigen CD172a as a specific marker for cardiomyocyte-derived EVs, both *in vitro* and *in vivo* experiments showed that hypoxia increases EV yield (110). Small extracellular vesicles circulating in ST-segment elevation myocardial infarction (STEMI) reflect the severity of myocardial injury in patients (111). By analyzing monocyte transcriptome data, a strong positive correlation was observed between PKIG and naïve B cells in AMI-monocyte-derived exosomes, RPL23 monocyte-derived exosomes showed a positive correlation, and OST4 potentially interacts with the p53 signaling pathway to mediate cardiomyocyte apoptosis (112). Through analysis of EVs in postmortem body fluids, significantly elevated levels of miR-486-5p were found in patients with high-grade atherosclerotic plaques, suggesting its potential as a biomarker for diagnosing acute myocardial infarction triggered by coronary atherosclerosis, including within the forensic field (113). Proteomic profiling of urinary EVs revealed reduced expression levels of UMOD protein in CAD patients' urinary EVs, this study contributes to the future use of uEVs as an emerging biomarker for the early, non-invasive diagnosis of CAD through protein differentials (114). Proteomic identification of plasma exosomes also revealed differences in the types and levels of protein expression in both STEMI and NSTEMI (115). Analysis of human plasma small extracellular vesicles (sEVs) using surface-enhanced Raman scattering (SERS) measurement technology combined with various machine learning methods demonstrated high sensitivity and specificity, suggesting that interdisciplinary fusion provides a promising strategy for non-invasive, safe, and high-precision early detection of CAD (116). Transcending the limitations of conventional MI biomarkers, EVs leverage their cellular origin specificity and molecular stability to redefine the paradigms of early warning, precise diagnosis, and dynamic monitoring in acute coronary syndromes through multi-omics dimensions.[Table T4]

**Table 4 T4:** EVs as biomarkers in CVDs (117–133).

EV Cargo	Source	Disease	Clinical outcomes	Ref.
lncRNA Neat1	Cardiomyocytes	Cardiac ischemia	lncRNA Neat1 EV modulates the expression of P53 target genes, cell-cycle regulators and promoted cellular survival.	Long noncoding RNA-enriched vesicles secreted by hypoxic cardiomyocytes drive cardiac fibrosis
lncRNA CoroMarker	Monocytes	CAD	CoroMarker was stable in plasma because it was mainly in the EVs which is a stable, sensitive and specific biomarker for CAD	Plasma long non-coding RNA, CoroMarker, a novel biomarker for diagnosis of coronary artery disease
miR-126	Plasma	CAD	Increased plasma EV miR-126 and miR-199a reduce the risk of major cardiovascular outcomes in CAD patients	MicroRNA expression in circulating microvesicles predicts cardiovascular events in patients with coronary artery disease
miR-199a				
miR-155	Urine	CAD	Increased urinary EV miR-155 as a biomarker of CAD progression	MicroRNA-155 is decreased during atherosclerosis regression and is increased in urinary extracellular vesicles during atherosclerosis progression
miR-92a	Endothelialium	CAD	EC-derived EV miR-92a is increased in CAD patients. miR-92a regulates angiogenesis in recipient EC	Atherosclerotic conditions promote the packaging of functional microRNA-92a-3p into endothelial microvesicles
miR-1a	Plasma	STEMI	In STEMI patients, miR-1, -133a, -133b, and -499-5p were up-regulated, where as miR-122 and -375 were already lower than in healthy subjects	Circulating microRNAs are new and sensitive biomarkers of myocardial infarction
miR-133a/b				
miR-499-5p				
miR-122				
miR–375				
miR-192	Plasma	AMI	The 3 p53-responsive miRNAs that were upregulated in the serum of post-AMI patients who experienced development of HF within 1 year of AMI onset	Circulating p53-responsive microRNAs are predictive indicators of heart failure after acute myocardial infarction
miR-194				
miR-34a				
miR-24	Plasma	CAD	The plasma concentrations of exosomes and their cargo of cardiac miRs increased in patients undergoing CABG and were positively correlated with hs-cTnI	Coronary artery-bypass-graft surgery increases the plasma concentration of exosomes carrying a cargo of cardiac MicroRNAs: an example of exosome trafficking out of the human heart with potential for cardiac biomarker discovery
miR-208a/b				
miR-122				
miR-210				
miR-9	Serum	AIS	serum exosomal miR-9 and miR-124 are promising biomarkers for diagnosing AIS and evaluating the degree of damage caused by ischemic injury	MiR-9 and MiR-124 in the serum exosomes of acute ischemic stroke patients
miR-124				
miR-134	Serum	AIS	RT-qPCR analysis showed that exosomal miR-134 was significantly increased in AIS patients within 24 h after stroke onset compared with that of control group	Increased serum exosomal miR-134 expression in the acute ischemic stroke patients
miR-152-3p	Serum	AIS	Serum exosome miR-152-3p in patients with AIS was significantly lower than in healthy controls	Decreased Serum Exosomal miR-152-3p Contributes to the Progression of Acute Ischemic Stroke
miR-21-5p	Plasma	IS	The plasma-derived exosomal miR-21-5p and miRNA-30a-5p in combination are promising biomarkers for diagnosing IS and distinguishing among HIS, SIS, and RIS, especially miRNA-30a-5p for the diagnosis of the HIS phase	Diagnosis of hyperacute and acute ischaemic stroke: the potential utility of exosomal MicroRNA-21-5p and MicroRNA-30a-5p
miR-30a-5p				
miR-422a	Plasma	IS	Plasma exosomal miR-422a and miR-125b-2-3p may serve as blood-based biomarkers for monitoring and diagnosing in IS patients, with plasma exosomal miR-422a showing the best diagnostic value	Plasma Exosomal miR-422a and miR-125b-2-3p serve as biomarkers for ischemic stroke
miR-125b-2-3p				
miR-223	Serum	AIS	The level of miR-223 in circulating exosomes was elevated after onset of acute ischemic stroke, stroke patients with poor outcomes inclined to have a greater exosomal miR-223 expression	Increased circulating exosomal miRNA-223 is associated with acute ischemic stroke
let-7b-5p	Plasma	AIS	In AIS patient, hsa-let-7b-5p, hsa-miR-16-5p, and hsa-miR-320c were upregulated, whereas hsa-miR-548a-3p and hsa-miR-6808-3p	Differentially expressed miRNA profiles of serum derived extracellular vesicles from patients with acute ischemic stroke
miR-16-5p				
miR-320c				
miR-548a-3p				
miR-6808-3p				
miR-140-5p	Plasma	AIS	Within the first 6 h of symptom onset, three specific miRNAs exhibited significant differential expression compared to other time points and healthy controls	MicroRNA expression profile in acute ischemic stroke
miR-210-3p				
miR-7-5p				
CD31/Annexin 5	Plasma	CAD	Increased plasma CD31/Annexin 5 EVs as an independent predictor of cardiovascular events in CAD patients	Circulating CD31+/Annexin V+ microparticles correlate with cardiovascular outcomes
C1Q1A	Plasma	Myocardial infarction	Plasma EV proteins as predictive biomarkers and therapeutic targets in myocardial infarction	Plasma-derived extracellular vesicles contain predictive biomarkers and potential therapeutic targets for Myocardial Ischemic (MI) injury
C5				
GP1BA				
PPBP				
APOD				
APOC3				
CD144	Plasma	Myocardial injury	Increased plasma of CD144-EVs as predictor of cardiovascular complications	Significance of a multiple biomarkers strategy including endothelial dysfunction to improve risk stratification for cardiovascular events in patients at high risk for coronary heart disease
CD144	Endothelialium	Myocardial infarction	Endothelium-derived microparticles can improve prediction of future cardiovascular events in patients at high risk for CHD	
mCRP	Monocytes	CAD	mCRP in monocyte-derived EVs as biomarker of inflammatory process in CAD patients	Crp is transported by monocytes and monocyte-derived exosomes in the blood of patients with coronary artery disease
mCRP	Endothelialium	Myocardial infarction	EV transport and delivery of pro-inflammatory mCRP in AMI patients	Serum extracellular vesicle protein levels are associated with acute coronary syndrome
mCRP	Endothelialium	PAD	EC-derived EV mCRP is increased in patients with PAD, and was suggested as a predictor of vascular disease risk	Plasma Levels of Endothelial Microparticles Bearing Monomeric C-reactive Protein are Increased in Peripheral Artery Disease
CD11b	Urine	CAD	Increased CD45+ and CD11b+ and decreased CD16+ in urinary EVs are associated with CAD progression	MicroRNA-155 Is Decreased During Atherosclerosis Regression and Is Increased in Urinary Extracellular Vesicles During Atherosclerosis Progression
CD16				
CD45				
Sphingolipid	Plasma	STEMI	EV lipid signature discriminates STEMI patients and may be used as therapeutic strategy	Analysis of urinary exosomal metabolites identifies cardiovascular risk signatures with added value to urine analysis

#### 3.3.3 Mechanisms of stem cell-derived EVs in MI treatment

The EV-based regenerative toolkit is reshaping post-injury myocardial functional restoration through synergistic engineered delivery strategies and endogenous repair mechanisms (Table 5). Hypoxia and tumor necrosis factor-alpha (TNF-α) modulate extracellular vesicle release from human induced pluripotent stem cell-derived cardiomyocytes, this research aids in developing novel pharmacological strategies for MI (77). Extracellular vesicles from pluripotent stem cells or specially processed endogenous human serum exert cardioprotective effects post-injury (134). Mesenchymal stem cell-derived EVs (MSC-EVs) can rescue myocardial I/R injury by inducing cardiomyocyte autophagy via the AMPK and Akt pathways, reducing apoptosis and myocardial infarct size while improving cardiac function (135). Culturing MSC-EVs using a hollow fiber bioreactor-based three-dimensional (3D) system yields higher quantities with similar biological functions compared to traditional two-dimensional (2D) culture (136). Monocyte mimics confer stronger recruitment characteristics to MSC-EVs in I/R models through Mac1/LFA1-ICAM-1 adhesion molecule interactions, enhancing their targeting efficiency to injured myocardium and improving EV delivery to ischemic-damaged myocardium (137). Exosomes derived from adipose stem cells (ASC-Exos) promote angiogenesis in ischemic hindlimbs and hearts in mice by delivering miR-31 via the miR-31/FIH1/hypoxia-inducible factor-1α (HIF-1α) signaling pathway, alleviating ischemic heart disease (138). ASC-Exos-delivered miR-205 significantly reduced cardiomyocyte apoptosis while promoting angiogenesis and microvascular endothelial cell proliferation to improve cardiac function after myocardial infarction (139). Adipose stem cell-derived exosomal miR-196a-5p and miR-425-5p prevented ischemia-induced mitochondrial dysfunction and reactive oxygen species (ROS) production in cardiomyocytes, increased angiogenesis, and polarized macrophages towards the anti-inflammatory M2 immunophenotype (140). Furthermore, miR-196a-5p reduces and reverses myofibroblast activation and lowers collagen expression to inhibit myocardial fibrosis. ASCs-EVs-miR-221 significantly enhanced proliferation and expression of anti-apoptotic proteins in H9C2 cells (a cardiomyocyte cell line derived from the left ventricle of Sprague Dawley rats) (141). Plasma-derived extracellular vesicles from myocardial infarction patients inhibited TNF-α-induced cardiomyocyte death by suppressing TNF-α expression. yREX3 alleviates myocardial ischemic injury through selective DNA methylation (142). The non-coding RNA yREX3 encapsulated within EVs mediates the epigenetic silencing of the protein interacting with C kinase 1 (Pick1) via methylation of upstream CpG sites by triggering widespread transcriptomic changes in macrophages. Simultaneously, yREX3 interacts with polypyrimidine tract-binding protein 3 (PTBP3) to methylate the Pick1 gene locus. Inhibiting Pick1 in macrophages enhances Smad3 signaling and boosts exocytosis, thereby minimizing cardiac necrosis in rats with myocardial infarction. The neonatal mammalian heart possesses the ability to regenerate after injury by inducing cardiomyocyte proliferation. Experiments demonstrated that EVs from regenerating neonatal mouse heart tissue after apical resection surgery (AR-Neo-EVs) exhibited stronger pro-proliferative, anti-apoptotic, and pro-angiogenic activities compared to EVs from neonatal mouse heart tissue (Neo-EVs) (143). Subsequent studies confirmed that delivering AR-Neo-EVs via sodium alginate hydrogel microspheres is an effective method for treating myocardial infarction. Conversely, vesicles derived from myocardial tissue of postnatal day 8 mice (P8-EVs) significantly promoted M1-like macrophage polarization, enhanced phagocytosis, and influenced macrophage factor secretion by activating the Pak2-Erk1/2 axis, thereby inhibiting neonatal cardiomyocyte proliferation. Human amniotic fluid stem cell-derived EVs demonstrated potential for cardiomyocyte renewal (144). It shows that stem cell derived EVs offer enhanced translational potential as delivery vehicles for myocardial regeneration therapy by improving cardiac targeting, coordinating multiple repair pathways, and preserving bioactive molecule stability[Table T5].

**Table 5 T5:** Potential treatment of EVs in MI (138, 139, 145–157).

Source	Cargos	Signaling pathway/target	Function	Reference title
Mesenchymal stem cell derived EVs	miR-210	Efna3↓	proangiogenic effects	Mesenchymal stem cells-derived extracellular vesicles, via miR-210, improve infarcted cardiac function by promotion of angiogenesis
	–	NF-κB↓ → Nrf2/HO-1↑	anti-inflammation and polarizing M2 macrophage	Exosomes secreted by FNDC5-BMMSCs protect myocardial infarction by anti-inflammation and macrophage polarization via NF-κB signaling pathway and Nrf2/HO-1 axis
	miR-223	P53/S100A9↓	relieved myocardial fibrosis and inflammation infiltration	Human umbilical cord mesenchymal stem cell-derived extracellular vesicles loaded with miR-223 ameliorate myocardial infarction through P53/S100A9 axis
	miR-212-5p	NLRC5/VEGF/TGF-beta 1/SMAD↓	attenuate MI/cardiac fibrosis	MSCs-Derived Extracellular Vesicles Carrying miR-212-5p Alleviate Myocardial Infarction-Induced Cardiac Fibrosis via NLRC5/VEGF/TGF-β1/SMAD Axis
Adipose stem cell derived EVs	miR-31	FIH1/HIF-1α↑	proangiogenic effects	Exosomes from adipose-derived stem cells alleviate myocardial infarction via microRNA-31/FIH1/HIF-1α pathway
	miR-205	HIF-1α/VEGF↑	proangiogenic effects	ADSC-derived exosomes attenuate myocardial infarction injury by promoting miR-205-mediated cardiac angiogenesis
	miR-24-3p	Nrf2↑	cardioprotective effects	Adipose stem cell-derived nanovesicles for cardioprotection: production and identification of therapeutic component
Induced pluripotent stem cell derived EVs	miR-106a-363	Jag1/Notch3/Hes1↓	myocardial repairment	miR-106a–363 cluster in extracellular vesicles promotes endogenous myocardial repair via Notch3 pathway in ischemic heart injury
	miR-145/let-7/miR-302a-5p	BMP-4/PDGFα/VEGF-C↑	proangiogenic effects	Induced Pluripotent Stem Cell (iPSC)–Derived Extracellular Vesicles Are Safer and More Effective for Cardiac Repair Than iPSCs
Hypoxia treated stem cell derived EVs	miR224-5p	TXNIP↓ → HIF-1α↑	protect myocardial cells from hypoxic vulnerability	Extracellular vesicles from hypoxia-preconditioned mesenchymal stem cells alleviates myocardial injury by targeting thioredoxin-interacting protein-mediated hypoxia-inducible factor-1α pathway
Blueberry-treated stem cell derived EVs	–	–	anti-inflammatory and anti-apoptotic effects	The Therapeutic Effects of Blueberry-Treated Stem Cell-Derived Extracellular Vesicles in Ischemic Stroke
Engineered stem cell derived EVs	p38α antagonistic peptides	Mapk14↓ → α-SMA/collagen I↑	Inhibitory effect on CFs fibrosis	Sodium Alginate Hydrogel Infusion of Bone Marrow Mesenchymal Stem Cell-Derived Extracellular Vesicles and p38α Antagonistic Peptides in Myocardial Infarction Fibrosis Mitigation
	miR-210-3p	EFNA3↓	promoted angiogenesis	Extracellular Vesicles from NMN Preconditioned Mesenchymal Stem Cells Ameliorated Myocardial Infarction via miR-210-3p Promoted Angiogenesis
	Melatonin	ROS↓	inhibiting oxidative stress	Melatonin Engineered Adipose-Derived Biomimetic Nanovesicles Regulate Mitochondrial Functions and Promote Myocardial Repair in Myocardial Infarction
Human umbilical cord mesenchymal stem cell-derived Evs	–	PI3 K/Akt↑	alleviate ER stress–induced apoptosis	Extracellular Vesicles Derived from Human Umbilical Cord Mesenchymal Stromal Cells Protect Cardiac Cells Against Hypoxia/Reoxygenation Injury by Inhibiting Endoplasmic Reticulum Stress via Activation of the PI3K/Akt Pathway

#### 3.3.4 Potential therapeutic effect of engineered EVs in MI

Source platelet lysate-derived EVs (SCPL-EVs) carry a range of trophic factors and multiple recognized cardioprotective miRNAs (85). Experiments demonstrated their protection of rodent and human cardiomyocytes from I/R injury, stimulation of angiogenesis in human cardiac microvascular endothelial cells, and reduction of scar formation, thereby improving cardiac function. miR-4496 and miR-4691-5p are highly enriched in human embryonic stem cell-derived EVs (hESC-eEVs) (158). Overexpression of miR-4496 or miR-4691-5p led to increased endothelial cell (EC) tube formation and wound closure *in vitro*, confirming the novel pro-angiogenic functions of these miRNAs. CD47 signaling can help evade macrophage clearance by binding to signal regulatory protein alpha (SIRPα) (159). Purified CD47-EVs were encapsulated with miR-21a (a specific anti-apoptotic miRNA) via electroporation to construct Electro-CD47-EVs. This approach effectively improved the biodistribution of these miR-21a-containing EVs in the heart, prolonged their retention time in circulation, and reduced phagocytic clearance, providing new insights for potential therapeutic tools for myocardial I/R injury. EVs were functionalized on their surface with cardiac-targeting peptides (CTP) via genetic modification to generate cardiac-targeted EVs (CTP-EVs). Curcumin was then loaded into these CTP-EVs (CTP-EV-Cur) to specifically deliver curcumin to the heart, giving it higher bioavailability and enhancing its cardioprotective efficiency (160). Interestingly, after validating that miR-144-3p is a primary contributor to the therapeutic effects mediated by curcumin, co-loading curcumin and miR-144-3p into the CTP-EVs (CTP-EVs-Cur) retained their active cardiac targeting ability while exhibiting stronger cardioprotective effects both *in vitro* and *in vivo*. Targeting miR-222-engineered extracellular vesicles (TeEVs), tailored with CTPs, are developed as ischemic TeEV therapeutics. These TeEVs are encapsulated within mechanical hydrogels to create injectable TeEV-loaded cardiac patches, enabling minimal invasiveness to attenuate IRI. The injectable patches facilitate the precise targeting of TeEVs for the efficient rescue of damaged cells. Persistent delivery of TeEVs into the infarcted region alleviates acute IRI and mitigated remodeling post IRI. This is linked to focal adhesion activation, cytoskeleton force enhancement, and nuclear force-sensing preservation. These findings may pave the way for force-sensing approaches to cardiac therapy using bioengineered therapeutic patches (161). Evidence demonstrates that engineered EVs via peptide-directed targeting, therapeutic cargo loading, and hydrogel-based delivery systems, significantly enhance cardiac drug accumulation, prolong circulation half-life, and enable sustained release in injured areas, offering novel precision strategies for myocardial I/R injury.

### 3.4 Mechanisms of EVs in heart failure and cardiac remodeling

Heart failure (HF) represents a complex clinical syndrome characterized by progressive cardiac remodeling following injury. Extracellular vesicles have emerged as pivotal mediators in this process, influencing pathological remodeling, serving as biomarker sources, and offering novel therapeutic opportunities. This section delineates the multifaceted roles of EVs in HF, spanning their contributions to disease mechanisms, diagnostic potential, and therapeutic applications.

#### 3.4.1 Mechanisms of EVs in HF damage and remodeling

Chronic heart failure (CHF) is associated with redox imbalance. In CHF induced after MI, EVs enriched with cardiac miRNAs can mediate enhanced oxidative stress and heightened cardiac and central sympathetic nerve excitation by targeting and downregulating the Nrf2/antioxidant signaling pathway (162). The miR-155-5p in heart-derived EVs promotes macrophage M1 polarization by activating the JAK2/STAT1 pathway, leading to local cardiac inflammation and even triggering systemic inflammation in distant organs (106). Conversely, some EVs exhibit protective properties. In EVs derived from human induced pluripotent stem cell-derived cardiomyocytes (hCM-EVs), transfection of human cardiac fibroblasts (hCFs) with miR-24-3p prevented TGF-β1-mediated induction of FURIN, CCND1, and SMAD4—miR-24-3p target genes involved in TGF-β1-dependent fibrosis—regulating the transformation of hCFs to myofibroblasts (163). Furthermore, the long noncoding RNA Tcf21 antisense RNA inducing demethylation (lncRNA-TARID), enriched in EVs, was found to upregulate Tcf21 expression, which suppresses transforming growth factor beta (TGF-β) signaling and myofibroblast differentiation to inhibit cardiac fibrosis (164). Circular RNA circWhsc1 was found enriched in neonatal mouse hearts, particularly in cardiac ECs, and further upregulated in ECs and EC-derived EVs under hypoxic conditions. EV-derived circWhsc1 activates TRIM59 by enhancing its phosphorylation, thereby strengthening TRIM59 binding to STAT3, phosphorylating STAT3, and inducing cardiomyocyte (CM) proliferation (165). These findings highlight the dual role of EVs in heart failure—mediating both detrimental processes such as oxidative stress, inflammation, and sympathetic overactivation, as well as protective mechanisms including anti-fibrotic and pro-regenerative effects—suggesting their potential as therapeutic targets in HF management.

#### 3.4.2 Diagnostic potential of EVs in HF

The molecular cargo of EVs provides a rich source for discovering biomarkers to monitor HF progression and predict outcomes. Temporal changes in the ratio of coagulation and fibrinolysis pathway proteins in low-density lipoprotein-associated EVs (LDL-EVs) may be more advantageous than currently used plasma biomarkers for predicting reverse left ventricular remodeling after acute myocardial infarction (AMI) (166). Furthermore, downregulated circCEBPZOS was detectable in serum EVs from patients with adverse cardiac remodeling. CircCEBPZOS acts as a competitive endogenous RNA (ceRNA) by directly binding miR-1178-3p, thereby promoting transcription of phosphoinositide-dependent kinase-1 (PDPK1), and its reduced levels are associated with deleterious post-MI remodeling and worse cardiac function (167). In summary, EVs offer a promising platform for the identification of novel biomarkers that reflect dynamic pathophysiological changes in HF, enabling early risk stratification, precise monitoring of disease progression, and improved prediction of clinical outcomes beyond conventional diagnostic tools.

#### 3.4.3 Therapeutic potential of EVs in HF

The therapeutic application of EVs, particularly from stem cells and engineered sources, holds significant promise for mitigating cardiac remodeling and improving function in HF. Stem cells from multiple sources offer potential for cardiac remodeling after myocardial infarction and heart failure. EVs from human umbilical cord mesenchymal stem cells (hUCMSCs) pre-treated with NMN, highly expressing miR-210-3p, significantly enhanced tube formation, migration, and proliferation capabilities in human umbilical vein endothelial cells (HUVECs) (153). N-EVs (NMN-pretreated EVs) promoted infarct healing through miR-210-3p targeting EFNA3 to improve angiogenesis. Hypoxia-induced adipose-derived stem cell EVs (ADSC-EVs) exhibited stronger proliferation, migration, and tube formation capabilities in HUVECs compared to normoxic ADSC-EVs and demonstrated enhanced neovascularization in mouse models (153). Intrapericardial injection of engineered EVs containing lncRNA-TARID upregulated Tcf21 expression in epicardium-derived cells and improved cardiac function and histology in mice and porcine MI models (164). In particular, milk EVs derived from colostrum contain various anti-inflammatory factors facilitating the transition from inflammation to proliferation phase, as well as factors for tissue remodeling and angiogenesis (168). Bovine milk-derived exosomes (BM-Exos) alleviate fibrosis through pro-angiogenic mechanisms, thereby improving cardiac fibrosis. In both *in vivo* models of isoproterenol (ISO)-induced cardiac fibrosis and *in vitro* OGD-treated HUVECs, BM-Exos were demonstrated to reduce extracellular matrix (ECM) deposition, improve cardiac function, and enhance pro-angiogenic growth factors (91).

Collectively, EVs play a dual role in heart failure, both as drivers of pathological remodeling and as vectors for innovative diagnostics and therapeutics. Understanding their intricate mechanisms and harnessing their potential paves the way for novel precision medicine approaches in cardiovascular disease.

### 3.5 Mechanisms of EVs in stroke

Acute ischemic stroke (AIS) is the most common type of stroke. Despite significant advances in stroke treatment, including but not limited to reperfusion therapies, the limited time window after stroke onset, the effectiveness of treatments, and associated risks highlight the necessity of researching novel diagnostic and therapeutic strategies.

#### 3.5.1 Mechanisms of EVs in stroke damage and repairment

Analogous to the peripheral vascular system, under hypoxic conditions, cerebral microvascular endothelial cell (CEC)-derived exosomes promote microglial M1 polarization and endothelial cell injury by delivering circ-0000495 (169). Conversely, ischemic CEC-derived small extracellular vesicles (CEC-sEVs), enriched in miR-27a, play a crucial role in the ischemic brain repair process by promoting axonal remodeling and improving neurological outcomes (170). sEVs derived from activated neurons, enriched in miR-100-5p, can activate the NF-κB pathway, causing abnormal activation of adjacent neurons and expanding the scope of neuronal damage, while also driving microglial activation to exacerbate neuroinflammation (171). Apoptosis induced by AIS releases substances that stimulate microglia to activate pro-inflammatory pathways and enhance EV production, or have the opposite effects. M2-polarized microglia-derived EVs, enriched in miR-124, reduce astrocyte proliferation by decreasing Signal Transducer and Activator of Transcription 3 (STAT3) gene signaling, thereby reducing glial scar formation and promoting post-stroke recovery (172). Furthermore, M2 microglia-derived small EVs, enriched in miR-93-5p and miR-25-3p, downregulated the expression of TGFBR, PTEN, and FOXO3 in neural stem cells (NSCs), increasing NSC proliferation and neuronal differentiation. Inhibition of the extracellular matrix protein Sema3A in astrocyte-derived EVs during the subacute phase of stroke suppresses activated astrocytes in the peri-infarct cortex and enhances neurological functional recovery (173). Exosomes secreted by ischemic astrocytes treated with a Sema3A inhibitor further increase Prostaglandin D2 Synthase (PTGDS) expression and contribute to post-stroke axonal growth and elongation, as well as functional recovery (174). In spontaneous intracerebral hemorrhage (ICH), red blood cell-derived microparticles inhibit hematoma growth in ICH (175). EVs in brain injury bidirectionally regulate neuroinflammation and regeneration via non-coding RNA delivery including endothelial and neuron-derived EVs exacerbate microglial polarization and excitotoxicity, whereas ischemia-adapted endothelial and glial vesicles promote axonal remodeling and suppress scarring.

#### 3.5.2 Diagnostic effect of EVs in stroke

To date, stroke diagnosis primarily relies on clinical symptom assessment and neuroimaging, suitable blood biomarkers for stroke detection via blood tests remain undeveloped. Extracellular vesicles (EVs), on the one hand, can cross the blood-brain barrier (BBB), and more importantly, reflect the real-time status of the secreting cells, thereby offering the possibility of serving as diagnostic biomarkers for brain diseases (Table 4). Examination of plasma EV markers (CD9+) alongside neuronal markers (L1CAM, CD171), astrocytic markers (EAAT1), and oligodendrocyte markers (MOG) demonstrated elevated levels of astrocyte-derived extracellular vesicles (ADEs) within the first month after stroke in clinical settings (176). Multiple studies have proposed that EVs derived from plasma, through analysis of their miRNA and proteomics, confirm their role in the pathophysiology of acute stroke and offer potential biomarkers for stroke diagnosis.

#### 3.5.3 Potential therapeutic effect of stem cell-derived EVs in stroke

After AIS, both bone marrow mesenchymal stem cell-derived EVs (BMSC-EVs) and brain endothelial cell-derived EVs (BEC-EVs) protect blood-brain barrier (BBB) integrity, with BMSC-EVs enhancing efficacy in improving neurological function. Both EVs attenuate BBB permeability by inhibiting the Cav-1/CD147/VEGFR2/MMP pathway and restore tight junction protein expression by counteracting abnormal endocytosis, repairing and enhancing BBB integrity (177). MSC-derived EVs in the treatment of ischemic cerebrovascular disease exhibit mechanisms and efficacy similar to the previously mentioned MSC-EVs in repairing vascular endothelium during myocardial ischemia. Adipose-derived MSCs, via their contained miR-760-3p, inhibit CHAC1 expression to suppress the progression of ferroptosis, a key process in ischemic stroke, effective delivery to the brain via intranasal (IN) administration alleviates cerebral ischemia/reperfusion injury (178). Dental pulp stem cell-derived EVs, enriched in miR-877-3p, interact with Bclaf1, reducing neuronal apoptosis, decreasing infarct size, and improving cerebral edema (179). Extracellular vesicles derived from blueberry-treated mesenchymal stem cells (MSCs) demonstrated significantly greater protection against neuronal cell death in oxygen-glucose deprivation (OGD) cells and middle cerebral artery occlusion (MCAo) animal models compared to the untreated group, both EV treatments increased cell viability, reduced cerebral infarction area in animal experiments, and improved behavioral assessments (151). EVs derived from Panax notoginseng, a commonly used Chinese herbal medicine, contain lipid components that suppress the inflammatory response by shifting the microglial phenotype from M1 to M2, alleviating cerebral ischemia/reperfusion injury (CI/R) (180). Furthermore, studies found that these lipids act on the PI3K/Akt pathway to reduce cerebral infarction volume and exert therapeutic effects. Momordica charantia (bitter melon) exosomes, via miR-5266, activate the AKT/GSK3β pathway, inhibit neuronal apoptosis in stroke, alleviate ischemia-reperfusion injury, and maintain BBB integrity (181). Application of NSC-derived exosomes (NSC-Exos) to an *in vitro* mouse neuronal injury model before and/or during OGD combined injury verified their significant protective effect on astrocytes. Mice treated with intravenously injected NSC-Exos after stroke and reperfusion injury all exhibited significantly reduced infarct volumes. On one hand, NSC-Exos promote neuroprotection by transferring miR-150-3p, which targets CASP2, inhibiting neuronal apoptosis after brain injury, on the other hand, they enhance the nuclear translocation of Nrf2 to counteract oxidative stress and reduce inflammatory responses (182). Simultaneously, NSC-Exos demonstrated functions promoting neuronal axon elongation and angiogenesis in HUVECs, offering a potential therapeutic avenue for AIS. Co-incubation of BDNF protein (a widely distributed neurotrophic factor) with hNSC-Exos for 24 h loaded BDNF into the exosomes, resulting in BDNF-hNSC-Exos, which exhibited stronger anti-apoptotic and pro-differentiation capabilities than hNSC-Exos. In a rat ischemic stroke model, BDNF-hNSC-Exos effectively suppressed microglial expression, inhibited inflammation, promoted endogenous NSC differentiation, thereby reducing infarct size and improving neurological function (80). Experimental data confirm that engineered extracellular vesicles subjected to molecular preconditioning, bioactive cargo loading, or delivery route optimization synergistically enhance barrier restoration, neuroprotection, and regenerative efficacy, with significant improvement in neurological outcomes in stroke models.

#### 3.5.4 Potential therapeutic effect of engineered EVs and PDEVs in stroke

Utilizing the property of bacterial-derived outer membrane vesicles (OMVs) to target neutrophils, pioglitazone (PGZ) was encapsulated into OMVs. The resulting OMV@PGZ nanoparticles inherited the functions associated with the bacterial outer membrane, facilitating targeted uptake by neutrophils (183). Results indicated OMV@PGZ enhanced PGZ delivery to the brain for treating ischemic stroke, while inhibiting nucleotide-binding oligomerization domain-like receptor protein 3 (NLRP3) inflammasome activation and ferroptosis, reducing inflammatory responses after reperfusion injury to exert neuroprotective effects. Experiments in tMCAO mice treated with Lactobacillus plantarum-derived EVs (LEVs) confirmed their efficacy in reducing apoptosis of ischemic neurons, potentially by inhibiting neuronal apoptosis via the miR-101a-3p/c-Fos/TGF-β axis, thereby preventing ischemia-induced brain injury (184). Engineered EVs in cerebrovascular diseases are mostly used to deliver therapeutic components. Utilizing the targeting ability of RGD peptide and the cell-penetrating ability of Angiopep-2 peptide, dual-modified adipose-derived stem cell EVs demonstrated high transcellular permeability across the BBB *in vitro*, while targeting ischemic blood vessels, *in vivo*, intravenous administration achieved rapid accumulation of active components in the ischemic lesion area. By modifying the surface of M2 microglia-derived EVs with rabies virus glycoprotein peptide 29 (RVG29), miR-221-3p and miR-423-3p within them exert anti-apoptotic effects on neurons via the p38/ERK signaling pathway in stroke (185). Momordica charantia-derived exosome-like nanoparticles (MC-ELNs) were demonstrated to cross the blood-brain barrier and accumulate in ischemic regions. They exerted neuroprotection by delivering miR-5266 to suppress matrix metalloproteinase 9 (MMP-9) expression which preserving BBB integrity and activating the AKT/GSK3β signaling pathway, ultimately reducing cerebral infarct volume and improving neurological deficits in MCAO rats (181). Researches show that engineered EVs and plant-derived EVs, via targeting modifications and therapeutic cargo loading, breach the blood-brain barrier for targeted delivery to cerebral ischemic foci, exerting neuroprotection through suppression of neuroinflammation and programmed cell death.

### 3.6 Mechanisms of EVs in valvular heart disease

Valvular heart disease (VHD) encompasses a range of structural and functional abnormalities of heart valves, leading to impaired blood flow. Extracellular vesicles play critical roles in the pathogenesis, diagnosis, and treatment of VHD, serving as key mediators of intercellular communication and potential vehicles for therapeutic intervention.

#### 3.6.1 Mechanisms of EVs in VHD pathogenesis

Valvular cells under stress or injury release EVs carrying a distinct molecular cargo that reflects the underlying pathophysiological processes. EVs derived from diseased valves exhibit increased levels of inflammatory cytokines, IL-6, TNF-α and calcification-promoting factors such as alkaline phosphatase, osteopontin, bone morphogenetic protein 2 (BMP2), and MMPs, which drive inflammation, calcification, and tissue remodeling in VHD (186). Additionally, dysregulation of specific miRNAs within EVs—such as downregulation of miR-143 and miR-145 which involved in valvular smooth muscle cell homeostasis and upregulation of miR-21, contributes to disease progression by promoting fibrosis and inhibiting apoptosis (187, 188). Lipidomic analyses further reveal elevated levels of ceramides, sphingomyelin, and oxidized phospholipids in EVs from diseased valves, which are associated with oxidative stress and apoptosis (189). EVs from calcified human aortic stenotic valves (AS-EVs) are highly enriched in TF and activate valvular endothelial cells via the AT1R/NADPH oxidase/SGLT2 pathway. This triggers a pronounced pro-oxidant, pro-inflammatory, and pro-thrombotic response, leading to endothelial dysfunction and heightened thrombogenicity, a process that can be mitigated by SGLT2 inhibition (190). Overall, these findings underscore that valvular-derived EVs are not merely bystanders but active contributors to the pathogenesis of valvular heart disease, driving a cascade of inflammatory, calcific, thrombotic, and fibrotic processes.

#### 3.6.2 Diagnostic potential of EVs in VHD

EVs offer a promising non-invasive approach for early detection and risk stratification of VHD. Their molecular cargo, including proteins, miRNAs, and lipids, provides a snapshot of valvular health and disease activity. For instance, EVs associated proteins such as MMPs and osteopontin can serve as early biomarkers before structural changes are detectable via echocardiography (191). miRNA signatures, such as miR-21, miR-143 and miR-145 within EVs can differentiate between VHD subtypes and predict disease progression (187). Multi-marker EV panels integrating protein, miRNA, and lipid profiles are under development to enhance diagnostic accuracy and prognostic value.

#### 3.6.3 Therapeutic potential of EVs in VHD

EVs hold significant promise as therapeutic agents in VHD, offering targeted and minimally invasive treatment strategies. Mesenchymal stem cell-derived EVs (MSC-EVs) exhibit potent anti-inflammatory effects by delivering anti-inflammatory cytokines (e.g., IL-10, TGF-β) and miRNAs (e.g., miR-146a), which attenuate valvular inflammation and fibrosis (192). Engineered EVs loaded with anti-calcific miRNAs (e.g., miR-302/367 cluster) can inhibit osteogenic differentiation of valvular interstitial cells and reduce calcification in preclinical models (193). EVs derived from telocytes (TCs) were shown to ameliorate calcific aortic valve disease (CAVD) in mice by delivering miR-30b, which targets Runx2 and inhibits the Wnt/β-catenin pathway, thereby reducing VICs osteogenic differentiation and calcium deposition (194). EVs derived from endothelial progenitor cells (EPCs) promote endothelial repair and angiogenesis, thereby mitigating fibrosis and improving valve function (195). Furthermore, EVs can be functionalized to deliver targeted therapeutics including statins and anti-fibrotic drugs directly to valvular tissue, enhancing treatment efficacy and reducing systemic side effects (196).

Collectively, EVs play dual roles in VHD, as mediators of disease progression and as innovative tools for diagnosis and therapy. Harnessing their potential through further research and clinical validation may lead to novel precision medicine approaches for valvular heart disease.

## 4 Challenges and development directions in the future

### 4.1 Technical hurdles

One of the primary challenges hindering the clinical application of EVs in CVDs is rooted in the technical limitations associated with their isolation, purification, and characterization. Owing to the inherent heterogeneity of EV populations, which are secreted by various cell types and exist in multiple subtypes such as exosomes, microvesicles, and apoptotic bodies, classical isolation methods often yield preparations that are contaminated with protein aggregates, lipoproteins, or other non-vesicular materials, thereby compromising the purity and, consequently, the reproducibility of downstream therapeutic applications (197).

Additional technical challenges involve limitations in cargo loading methodologies. The efficient incorporation of therapeutic molecules—such as specific miRNAs, proteins, or drugs—into EVs demands innovative strategies that preserve vesicle integrity while ensuring sufficient encapsulation efficiency. While techniques like passive incubation, electroporation, and sonication have been employed, each exhibits intrinsic drawbacks including cargo aggregation, potential vesicle disruption, and low loading efficiency, collectively constraining their utility. Critically, these challenges compound broader technical barriers spanning from the reproducible isolation of homogeneous EV populations to the standardization of characterization methods essential for evaluating therapeutic efficacy and pharmacokinetics. Collectively, these hurdles represent a fundamental impediment to developing reliable EV-based cardiovascular therapeutics.

#### 4.1.1 Limitations of EV-based biomarkers in CVDs

Biological variability presents further obstacles. Fluctuations in EV molecular cargo driven by physiological states including circadian rhythms or exercise, and comorbidities such as diabetes or renal dysfunction, obscure disease-specific signatures (198, 199). This instability is intensified by inconsistent pre-analytical variables, particularly variations in sample collection protocols, storage conditions, and processing delays across research cohorts. Critical validation deficiencies hinder clinical implementation (200). Most proposed EV biomarkers emerge from small discovery cohorts without external validation through multicenter trials. Longitudinal evidence demonstrating prognostic utility in diverse populations remains scarce, limiting risk stratification capabilities. Furthermore, the significant cost and technical demands of EV profiling compared to conventional biomarkers create economic barriers. Regulatory pathways for EV-based diagnostics are currently undefined. These collective challenges impede the translation of EV biomarker research into cardiovascular clinical practice (201).

### 4.2 Clinical trials involving EVs in CVDs

Over the last five years, EVs have emerged as promising biomarkers and therapeutic agents in cardiovascular disease owing to their ability to reflect the state of the parent cell and modulate regenerative processes. Their potential has been investigated across a wide spectrum of CVDs such as myocardial infarction, heart failure, ischemia/reperfusion injury, stroke, and atherosclerosis. A growing number of clinical trials have been initiated to explore the translational value of EVs.

A detailed analysis of the current clinical landscape, as summarized in Table 6, reveals several key trends. First, research on stroke, particularly acute ischemic stroke, constitutes a predominant focus of registered EV clinical trials in the CVDs field (e.g., NCT05877016, NCT04202783, NCT05191381). This highlights the urgent clinical need for novel diagnostic and therapeutic strategies in neurovascular diseases. Second, the majority of these registered studies are in early phases (I or II) and are primarily observational in nature. These trials aim to correlate specific EV signatures (e.g., surface proteins, miRNA content) with disease diagnosis, severity, or prognosis outcomes, laying the groundwork for EV-based liquid biopsies.

**Table 6:** Clinical trials involving EVs in CVDs.

Disease	Source	Meanings	Reference
Myocardial Infarction	Exosomes	Determine the miRNA expression profile in peripheral blood exosomes of patients with myocardial infarction	NCT04127591
Myocardial Infarction	MSC-Exosomes	Protect the heart against the harmful effects of ischemia and also IR, preserve cardiac function, reduce the zone of MI	NCT05669144
Acute Ischemic Stroke	MSC-Exosomes	Improves functional recovery and enhances neurite remodeling, neurogenesis, and angiogenesis after AIS	NCT03384433
Acute Ischemic Stroke	GD-iExo-003	Explore the effect of exosomes derived from HIPSC in AIS	NCT06138210
Acute Ischemic Stroke	MSC-Exosomes	Safety of cultured allogeneic adult umbilical cord derived MSC Exosomes intranasal instillations for the treatment of stroke	NCT05158101
Acute Ischemic Stroke	Exosomes	Characterization of circulating EVs/Exosomes as new predictive biomarkers in Stroke patients	NCT05370105
Acute Ischemic Stroke	RBC-Evs	RBC deformability will serve as a biomarker of the conditioning response and predictor of the clinical outcome in stroke patients	NCT04266639
Acute Ischemic Stroke	NSC-EVs	Clinical trial to assess the safety and efficacy of NouvSoma001 in the treatment of ischemic stroke	NCT06612710
Acute Ischemic Stroke	EVs	EVs profiles may change during acute stroke, in the chronic stroke phase, and according to the level of cSVD, may act as disease biomarkers	NCT06257823
Acute Ischemic Stroke	Serum EVs	Evaluation of predictive circulating biomarkers for rehabilitation recovery in ischemic and haemorrhagic stroke patients	NCT06871800
Stroke	EVs	Investigating the association of EVs and dysregulated coagulation in the prediction of Stroke	NCT05645081
Heart Failure	EVs	Investigate the expression of EVs RNAs during admission for and decongestion of ADHF patients and determine their diagnostic and prognostic potential	NCT06169540
Atherosclerosis	Fat tissue EVs	Research about EVs signaling in obesity and cardiometabolic diseases	NCT06408961
Hypertension	Urinary-Exosomes	Determine the concentrations and variabilities of urinary exosomal sodium channels and plasma angiotensins in patients with difficult-to-treat arterial hypertension and to investigate their dependency on clinical parameters and sampling conditions	NCT03034265
Hypertension	Endothelial-EVs	Assessing the prognostic significance of endotehlial dysfunction in hypertension	NCT06316271
Heart Failure	Plasma-Exosomes	Further explore the treatment's impact on heart failure-related inflammatory markers	NCT06501768
Atrial Fibrillation	Fat tissue EVs	Investigate the role of epicardial fat derived exosomes in patients who suffer from atrial fibrillation	NCT03478410
Heart Surgery	Blood MVs/Exosomes	Study of role of blood microvescicles and exosomes in patients with graft occlusion after aortocoronary bypass surgery	NCT05411445

Regarding the studied populations, most trials enroll patients with acute ischemic stroke, post-myocardial infarction, or chronic heart failure, reflecting the focus on acute events and subsequent maladaptive remodeling. In terms of interventional strategies, the few existing therapeutic trials mainly investigate the safety and efficacy of allogeneic MSC-EVs. For instance, the NCT05191381 trial employs intravenous infusion of allogeneic MSC-EVs for ischemic stroke treatment, while the NCT04327635 trial explores the intramyocardial injection of MSC-EVs to treat heart failure following myocardial infarction. In contrast, interventional clinical trials specifically targeting atherosclerosis itself remain scarce, indicating a significant gap and a future direction for translating mechanistic insights into therapeutic applications.

### 4.3 Difficulties in clinician translational

Beyond technical challenges, significant difficulties in translational medicine impede the clinical implementation of extracellular vesicle (EV)-based therapies for cardiovascular conditions. A pivotal aspect of translation is the scalability of EV production, inherently linked to the donor cell source and culture conditions. Many preclinical studies employ mesenchymal stem cells (MSCs) or cardiac progenitor cells to harvest EVs. However, these cells exhibit variable secretion rates, and the EV yield is often insufficient for human therapeutic dosing, necessitating the development of bioreactor-based systems and optimized cell expansion protocols. This lack of scalability is compounded by the difficulty in ensuring batch-to-batch consistency in EV composition and bioactivity, which remains sensitive to factors such as donor cell condition, culture medium, and processing variations.

Another major translational hurdle concerns the vivo biodistribution, clearance, and targeted delivery of EVs. Intravenously administered EVs are frequently cleared rapidly by the mononuclear phagocyte system (MPS), leading to suboptimal accumulation at intended cardiovascular sites. This is partly due to nonspecific uptake by organs like the liver, spleen, and lungs. Consequently, high dosages are required, which may be infeasible from a manufacturing standpoint and unacceptable from a safety perspective, especially given potential off-target effects. Furthermore, the dosing paradigms for EVs therapeutics remain inadequately defined; the effects of administered EV concentrations are influenced not only by the absolute dose but also by the interplay between EV stability, tissue targeting, and payload diversity. Due to their natural biodistribution, selective homing of EVs to diseased cardiovascular tissues remains suboptimal, and engineering modifications to enhance targeting have not yet yielded consistent improvements in *in vivo* delivery efficiency.

Safety concerns are also paramount. Despite the reduced immunogenicity of exosome-based therapies relative to cell-based approaches, potential risks persist, including the inadvertent transfer of oncogenic or pro-inflammatory genetic material, immunomodulatory effects that could provoke adverse reactions, and the possibility of horizontal gene transfer, necessitating rigorous long-term safety evaluations.

### 4.4 Frontier exploration

In response to the aforementioned challenges, recent research (primarily from the past five years) has focused on revolutionizing EV-based cardiovascular therapies through bioengineering and advanced technology integration. A key frontier involves enhancing EVs targeting specificity, cargo efficiency, and stability. Researchers now conjugate targeting ligands (e.g., peptides, antibodies) to direct EVs to damaged cardiac tissues, improving cardiomyocyte uptake while minimizing off-target effects. Notably, cardiac homing peptides show enhanced retention in infarcted myocardium in preclinical models (160). Parallel strategies employ rabies virus glycoprotein (RVG) antigens to achieve brain-directed EVs delivery, demonstrating the versatility of this targeting approach (185). Genetic and metabolic engineering further enable cargo customization with cardioprotective miRNAs/mRNAs to stimulate angiogenesis (202).

Advances in imaging represent another critical frontier. State-of-the-art modalities (e.g., PET/MRI radiolabeling) track EVs biodistribution and clearance kinetics *in vivo*, while live-cell reporters enable real-time monitoring (203). These technologies elucidate EVs pharmacokinetics and validate targeting efficacy under physiological conditions. Innovative biomaterial integration overcomes systemic delivery limitations. Incorporating EVs into hydrogels or cardiac patches sustains localized release at myocardial sites, improving bioavailability and leveraging biomaterial mechanical properties for tissue repair. Concurrently, synthetic EVs mimetics offer tunable composition and scalability for future therapies. Multi-omics and machine learning herald a new era: Integrated proteomic/transcriptomic/lipidomic profiling deciphers EVs molecular signatures from cardiac cells, identifying novel CVDs biomarkers and therapeutic targets. This facilitates precision medicine applications where individualized EVs profiles guide patient-specific interventions.

## 5 Conclusion

Cardiovascular diseases (CVDs) represent a persistent global health challenge, demanding innovative approaches for diagnosis and therapy. This review consolidates the multifaceted roles of extracellular vesicles (EVs) in CVDs, highlighting their dual capacity to propagate pathological processes while orchestrating tissue repair. Critically, EVs derived from diverse sources—including circulating biofluids, stem cells, and plants which carry unique molecular signatures that enable their utility as dynamic biomarkers for early detection and risk stratification. The emergence of engineered EVs and plant-derived EVs further expands their therapeutic potential, offering targeted strategies for myocardial and cerebrovascular injury. However, clinical translation requires overcoming challenges in standardization of isolation protocols, scalable production, and precise tissue delivery. Future research must integrate multi-omics technologies with advanced bioengineering platforms to unlock the full potential of EVs in precision cardiology.

## Glossary

AMI: acute myocardial infarction
AS: atherosclerosis
ASC: adipose stem cell
CEC: cerebral microvascular endothelial cell
CFs: cardiac fibroblasts
CHD: coronary heart disease
CI/R: cerebral ischemia/reperfusion injury
CM: cardiomyocyte
CTP: cardiac-targeting peptides
CVDs: cardiovascular diseases
EC: endothelial cell
eNOS: endothelial nitric oxide synthase
ESCRT: endosomal sorting complexes required for transport
ESTP: elastin-specific targeting peptide
EVs: extracellular vesicles
EXO: exosomes
HF: heart failure
HIF-1α: hypoxia-inducible factor-1α
I/R: ischemia-reperfusion injury
ICH: intracerebral hemorrhage
IHD: ischemic heart disease
lncRNA: long non-coding RNA
MI: myocardial infarction
miR: microRNA
MMP: matrix metalloproteinase
MPS: mononuclear phagocyte system.
MRI: magnetic resonance imaging
MSC: mesenchymal stem cell
MV: microvesicles
MVB: multivesicular bodies
NF-κB: nuclear factor kappa B
NMN: nicotinamide mononucleotide
NSC: neural stem cell
OGD: oxygen-glucose deprivation
Ox-LDL: oxidized low-density lipoprotein
P-EXO: platelet-derived exosomes
PDEVs: plant-derived EVs
PET: positron emission tomography
PS: phosphatidylserine
psEVs: plasma small extracellular vesicles
ROCK: rho-associated coiled-coil containing kinases
ROS: reactive oxygen species
RVG: rabies virus glycoprotein peptide
SERS: surface-enhanced Raman scattering
sEVs: serum EVs
STEMI: ST-segment elevation myocardial infarction
TF: tissue factor
TGF-β: tumor growth factor β
TNF-α: tumor necrosis factor α
Treg: regulatory T cell
VEGF: vascular endothelial growth factor
VSMC: vascular smooth muscle cell

## References

[1] Martin SS Aday AW Allen NB Almarzooq ZI Anderson CAM Arora P 2025 Heart disease and stroke statistics: a report of US and global data from the American Heart Association. Circulation. (2025) 151(8):e41–e660. 10.1161/CIR.000000000000001303 39866113 PMC12256702

[2] Li H Wang L Cheng H Zhang Q Wang S Zhong W Unlocking the potential of extracellular vesicles in cardiovascular disease. J Cell Mol Med. (2025) 29(3):e70407. 10.1111/jcmm.70407 39910696 PMC11798870

[3] Mosquera-Heredia MI Morales LC Vidal OM Barceló E Silvera-Redondo C Vélez JI Exosomes: potential disease biomarkers and new therapeutic targets. Biomedicines. (2021) 9(8):1061. 10.3390/biomedicines9081061 34440265 PMC8393483

[4] van Niel G D'Angelo G Raposo G. Shedding light on the cell biology of extracellular vesicles. Nat Rev Mol Cell Biol. (2018) 19(4):213–28. 10.1038/nrm.2017.125 29339798

[5] Akers JC Gonda D Kim R Carter BS Chen CC. Biogenesis of extracellular vesicles (EV): exosomes, microvesicles, retrovirus-like vesicles, and apoptotic bodies. J Neuro-Oncol. (2013) 113(1):1–11. 10.1007/s11060-013-1084-8 PMC5533094 23456661

[6] Clancy JW Schmidtmann M D’Souza-Schorey C. The ins and outs of microvesicles. FASEB Bioadv. (2021) 3(6):399–406. 10.1096/fba.2020-00127 34124595 PMC8171306

[7] Ratajczak MZ Ratajczak J. Extracellular microvesicles/exosomes: discovery, disbelief, acceptance, and the future? Leukemia. (2020) 34(12):3126–35. 10.1038/s41375-020-01041-z 32929129 PMC7685969

[8] Valadi H Ekström K Bossios A Sjöstrand M Lee JJ Lötvall JO. Exosome-mediated transfer of mRNAs and microRNAs is a novel mechanism of genetic exchange between cells. Nat Cell Biol. (2007) 9(6):654–9. 10.1038/ncb1596 17486113

[9] Shao HL Im H Castro CM Breakefield X Weissleder R Lee HH. New technologies for analysis of extracellular vesicles. Chem Rev. (2018) 118(4):1917–50. 10.1021/acs.chemrev.7b00534 29384376 PMC6029891

[10] Couzin J. Cell biology: the ins and outs of exosomes. Science. (2005) 308(5730):1862–3. 10.1126/science.308.5730.1862 15976285

[11] Gurung S Perocheau D Touramanidou L Baruteau J. The exosome journey: from biogenesis to uptake and intracellular signalling. Cell Commun Signal. (2021) 19(1):47. 10.1186/s12964-021-00730-1 33892745 PMC8063428

[12] Zhao J Zhu W Mao Y Li X Ling G Luo C Unignored intracellular journey and biomedical applications of extracellular vesicles. Adv Drug Deliv Rev. (2024) 212:115388. 10.1016/j.addr.2024.115388 38969268

[13] Kowal J Arras G Colombo M Jouve M Morath JP Primdal-Bengtson B Proteomic comparison defines novel markers to characterize heterogeneous populations of extracellular vesicle subtypes. Proc Natl Acad Sci U S A. (2016) 113(8):E968–77. 10.1073/pnas.1521230113 26858453 PMC4776515

[14] Santavanond JP Rutter SF Atkin-Smith GK Poon IKH. Apoptotic bodies: mechanism of formation, isolation and functional relevance. Subcell Biochem. (2021) 97:61–88. 10.1007/978-3-030-67171-6_4 33779914

[15] Yu L Zhu G Zhang Z Yu Y Zeng L Xu Z Apoptotic bodies: bioactive treasure left behind by the dying cells with robust diagnostic and therapeutic application potentials. J Nanobiotechnol. (2023) 21(1):218. 10.1186/s12951-023-01969-1 PMC10337089 37434199

[16] Yu W Yin N Yang Y Xuan C Liu X Liu W Rescuing ischemic stroke by biomimetic nanovesicles through accelerated thrombolysis and sequential ischemia-reperfusion protection. Acta Biomater. (2022) 140:625–40. 10.1016/j.actbio.2021.12.009 34902617

[17] Yoon JK Kim DH Kang ML Jang HK Park HJ Lee JB Anti-atherogenic effect of stem cell nanovesicles targeting disturbed flow sites. Small. (2020) 16(16):e2000012. 10.1002/smll.202000012 32239653

[18] Szatanek R Baj-Krzyworzeka M Zimoch J Lekka M Siedlar M Baran J. The methods of choice for extracellular vesicles (EVs) characterization. Int J Mol Sci. (2017) 18(6):1153. 10.3390/ijms18061153 28555055 PMC5485977

[19] Wiedmer SK Multia E Liangsupree T Riekkola ML. Automated on-line isolation and fractionation method for subpopulations of extracellular vesicles. Methods Mol Biol. (2023) 2668:99–108. 10.1007/978-1-0716-3203-1_8 37140792

[20] Welsh JA Arkesteijn GJA Bremer M Cimorelli M Dignat-George F Giebel B A compendium of single extracellular vesicle flow cytometry. J Extracell Vesicles. (2023) 12(2):e12299. 10.1002/jev2.12299 36759917 PMC9911638

[21] Yang J Gao X Xing X Huang H Tang Q Ma S An isolation system to collect high quality and purity extracellular vesicles from serum. Int J Nanomed. (2021) 16:6681–92. 10.2147/IJN.S328325 PMC8487857 34616151

[22] Xu Y Qin S An T Tang Y Huang Y Zheng L. MiR-145 detection in urinary extracellular vesicles increase diagnostic efficiency of prostate cancer based on hydrostatic filtration dialysis method. Prostate. (2017) 77(10):1167–75. 10.1002/pros.23376 28617988

[23] Vaswani K Koh YQ Almughlliq FB Peiris HN Mitchell MD. A method for the isolation and enrichment of purified bovine milk exosomes. Reprod Biol. (2017) 17(4):341–8. 10.1016/j.repbio.2017.09.007 29030127

[24] Zeng YB Deng X Shen LS Yang Y Zhou X Ye L Advances in plant-derived extracellular vesicles: isolation, composition, and biological functions. Food Funct. (2024) 15(23):11319–41. 10.1039/D4FO04321A 39523827

[25] Abeysinghe P Turner N Mitchell MD. A comparative analysis of small extracellular vesicle (sEV) micro-RNA (miRNA) isolation and sequencing procedures in blood plasma samples. Extracell Vesicles Circ Nucl Acids. (2024) 5(1):119–37. 10.20517/evcna.2023.55 39698410 PMC11648519

[26] Teixeira-Marques A Monteiro-Reis S Montezuma D Lourenço C Oliveira MC Constâncio V Improved recovery of urinary small extracellular vesicles by differential ultracentrifugation. Sci Rep. (2024) 14(1):12267. 10.1038/s41598-024-62783-9 38806574 PMC11133306

[27] Iwai K Minamisawa T Suga K Yajima Y Shiba K. Isolation of human salivary extracellular vesicles by iodixanol density gradient ultracentrifugation and their characterizations. J Extracell Vesicles. (2016) 5:30829. 10.3402/jev.v5.30829 27193612 PMC4871899

[28] Sidhom K Obi PO Saleem A. A review of exosomal isolation methods: is size exclusion chromatography the best option? Int J Mol Sci. (2020) 21(18):6466. 10.3390/ijms21186466 32899828 PMC7556044

[29] Yuan R Zhou Y Arias GF Dittmer DP. Extracellular vesicle isolation by a tangential-flow filtration-based large-scale purification method. Methods Mol Biol. (2023) 2668:45–55. 10.1007/978-1-0716-3203-1_5 37140789

[30] Valle-Tamayo N Pérez-González R Chiva-Blanch G Belbin O Serrano-Requena S Sirisi S Enrichment of astrocyte-derived extracellular vesicles from human plasma. J Vis Exp. (2022) (186):e64107. 10.3791/64107 35993755

[31] Brambilla D Sola L Ferretti AM Chiodi E Zarovni N Fortunato D EV separation: release of intact extracellular vesicles immunocaptured on magnetic particles. Anal Chem. (2021) 93(13):5476–83. 10.1021/acs.analchem.0c05194 33769802

[32] Chen J Zheng M Xiao Q Wang H Chi C Lin T Recent advances in microfluidic-based extracellular vesicle analysis. Micromachines (Basel). (2024) 15(5):630. 10.3390/mi15050630 38793203 PMC11122811

[33] Wu B Chen X Wang J Qing X Wang Z Ding X Separation and characterization of extracellular vesicles from human plasma by asymmetrical flow field-flow fractionation. Anal Chim Acta. (2020) 1127:234–45. 10.1016/j.aca.2020.06.071 32800129

[34] Su B Jeyhani M Thillainadesan G Sheng M Wunsche R Dayarathna T Next generation aqueous two-phase system for gentle, effective, and timely extracellular vesicle isolation and transcriptomic analysis. J Extracell Vesicles. (2025) 14(3):e70058. 10.1002/jev2.70058 40108918 PMC11923243

[35] Nieuwland R Siljander PR. A beginner’s guide to study extracellular vesicles in human blood plasma and serum. J Extracell Vesicles. (2024) 13(1):e12400. 10.1002/jev2.12400 38193375 PMC10775135

[36] Zhang X Takeuchi T Takeda A Mochizuki H Nagai Y. Comparison of serum and plasma as a source of blood extracellular vesicles: increased levels of platelet-derived particles in serum extracellular vesicle fractions alter content profiles from plasma extracellular vesicle fractions. PLoS One. (2022) 17(6):e0270634. 10.1371/journal.pone.0270634 35749554 PMC9231772

[37] Zeng Q Li W Luo Z Zhou H Duan Z Xiong XL. The role of miR1 and miR133a in new-onset atrial fibrillation after acute myocardial infarction. BMC Cardiovasc Disord. (2023) 23(1):448. 10.1186/s12872-023-03462-x 37697243 PMC10496401

[38] Seegobin N Taub M Vignal C Waxin C Chris V Awad A Small milk-derived extracellular vesicles: suitable vehicles for oral drug delivery? Eur J Pharm Biopharm. (2025) 212:114744. 10.1016/j.ejpb.2025.114744 40355010

[39] Salehi M Negahdari B Mehryab F Shekari F. Milk-derived extracellular vesicles: biomedical applications, current challenges, and future perspectives. J Agric Food Chem. (2024) 72(15):8304–31. 10.1021/acs.jafc.3c07899 38587896

[40] Aminzadeh MA Fournier M Akhmerov A Jones-Ungerleider KC Valle JB Marbán E. Casein-enhanced uptake and disease-modifying bioactivity of ingested extracellular vesicles. J Extracell Vesicles. (2021) 10(3):e12045. 10.1002/jev2.12045 33456725 PMC7798403

[41] Tiwari A Soni N Dongre S Chaudhary M Bissa B. The role of plant-derived extracellular vesicles in ameliorating chronic diseases. Mol Biol Rep. (2025) 52(1):360. 10.1007/s11033-025-10466-7 40180626

[42] Kim M Jang H Kim W Kim D Park JH. Therapeutic applications of plant-derived extracellular vesicles as antioxidants for oxidative stress-related diseases. Antioxidants (Basel). (2023) 12(6):1286. 10.3390/antiox12061286 37372016 PMC10295733

[43] Tsimikas S Witztum JL. Oxidized phospholipids in cardiovascular disease. Nat Rev Cardiol. (2024) 21(3):170–91. 10.1038/s41569-023-00937-4 37848630

[44] Yu F Duan Y Liu C Huang H Xiao X He Z. Extracellular vesicles in atherosclerosis and vascular calcification: the versatile non-coding RNAs from endothelial cells and vascular smooth muscle cells. Front Med (Lausanne). (2023) 10:1193660. 10.3389/fmed.2023.1193660 37469665 PMC10352799

[45] He C Kim HI Park J Guo J Huang W. The role of immune cells in different stages of atherosclerosis. Int J Med Sci. (2024) 21(6):1129–43. 10.7150/ijms.94570 38774746 PMC11103388

[46] Xu S Ilyas I Little PJ Li H Kamato D Zheng X Endothelial dysfunction in atherosclerotic cardiovascular diseases and beyond: from mechanism to pharmacotherapies. Pharmacol Rev. (2021) 73(3):924–67. 10.1124/pharmrev.120.000096 34088867

[47] Chen G Pei Y Jiang P Ye Q Xie Z Gyawali L. Exosomal NEDD4l derived from HG+oxLDL-induced vascular endothelial cells accelerates macrophage M1 polarization and oxLDL uptake by ubiquitinating IκBα and PPARγ. Cell Biol Toxicol. (2025) 41(1):23. 10.1007/s10565-024-09973-3 39775116 PMC11706887

[48] Taguchi K Hida M Narimatsu H Matsumoto T Kobayashi T. Glucose and angiotensin II-derived endothelial extracellular vesicles regulate endothelial dysfunction via ERK1/2 activation. Pflugers Arch. (2017) 469(2):293–302. 10.1007/s00424-016-1926-2 27975141

[49] Choi YY Kim A Lee Y Lee YH Park M Shin E The miR-126-5p and miR-212-3p in the extracellular vesicles activate monocytes in the early stage of radiation-induced vascular inflammation implicated in atherosclerosis. J Extracell Vesicles. (2023) 12(5):e12325. 10.1002/jev2.12325 37140946 PMC10158827

[50] Pham TT Le AH Dang CP Chong SY Do DV Peng B Endocytosis of red blood cell extracellular vesicles by macrophages leads to cytoplasmic heme release and prevents foam cell formation in atherosclerosis. J Extracell Vesicles. (2023) 12(8):e12354. 10.1002/jev2.12354 37553837 PMC10410060

[51] Bhattacharya P Dhawan UK Hussain MT Singh P Bhagat KK Singhal A Efferocytes release extracellular vesicles to resolve inflammation and tissue injury via prosaposin-GPR37 signaling. Cell Rep. (2023) 42(7):112808. 10.1016/j.celrep.2023.112808 37436891

[52] Bouchareychas L Duong P Covarrubias S Alsop E Phu TA Chung A Macrophage exosomes resolve atherosclerosis by regulating hematopoiesis and inflammation via MicroRNA cargo. Cell Rep. (2020) 32(2):107881. 10.1016/j.celrep.2020.107881 32668250 PMC8143919

[53] Wang C Liu C Shi J Li H Jiang S Zhao P Nicotine exacerbates endothelial dysfunction and drives atherosclerosis via extracellular vesicle-miRNA. Cardiovasc Res. (2023) 119(3):729–42. 10.1093/cvr/cvac140 36006370

[54] Bao H Li ZT Xu LH Su TY Han Y Bao M Platelet-derived extracellular vesicles increase Col8a1 secretion and vascular stiffness in intimal injury. Front Cell Dev Biol. (2021) 9:641763. 10.3389/fcell.2021.641763 33738288 PMC7960786

[55] Pan Y Liang H Liu H Li D Chen X Li L Platelet-secreted microRNA-223 promotes endothelial cell apoptosis induced by advanced glycation end products via targeting the insulin-like growth factor 1 receptor. J Immunol. (2014) 192(1):437–46. 10.4049/jimmunol.1301790 24307738

[56] Chen L Hu L Li Q Ma J Li H. Exosome-encapsulated miR-505 from ox-LDL-treated vascular endothelial cells aggravates atherosclerosis by inducing NET formation. Acta Biochim Biophys Sin (Shanghai). (2019) 51(12):1233–41. 10.1093/abbs/gmz123 31768526

[57] Li P Hong J Liang C Li Y Gao L Wu L Endothelial cell-released extracellular vesicles trigger pyroptosis and vascular inflammation to induce atherosclerosis through the delivery of HIF1A-AS2. FASEB J. (2023) 37(6):e22942. 10.1096/fj.202201399RRR 37178006

[58] Zhang Z Yi D Zhou J Zheng Y Gao Z Hu X Exosomal LINC01005 derived from oxidized low-density lipoprotein-treated endothelial cells regulates vascular smooth muscle cell phenotypic switch. Biofactors. (2020) 46(5):743–53. 10.1002/biof.1665 32663367

[59] Togliatto G Dentelli P Rosso A Lombardo G Gili M Gallo S PDGF-BB carried by endothelial cell-derived extracellular vesicles reduces vascular smooth muscle cell apoptosis in diabetes. Diabetes. (2018) 67(4):704–16. 10.2337/db17-0371 29386225

[60] Li K Cui M Zhang K Wang G Zhai S. M1 macrophages-derived extracellular vesicles elevate microRNA-185-3p to aggravate the development of atherosclerosis in ApoE(−/−) mice by inhibiting small mothers against decapentaplegic 7. Int Immunopharmacol. (2021) 90:107138. 10.1016/j.intimp.2020.107138 33302032

[61] Wang Q Dong Y Wang H. microRNA-19b-3p-containing extracellular vesicles derived from macrophages promote the development of atherosclerosis by targeting JAZF1. J Cell Mol Med. (2022) 26(1):48–59. 10.1111/jcmm.16938 34910364 PMC8742201

[62] Gao W Liu H Yuan J Wu C Huang D Ma Y Exosomes derived from mature dendritic cells increase endothelial inflammation and atherosclerosis via membrane TNF-α mediated NF-κB pathway. J Cell Mol Med. (2016) 20(12):2318–27. 10.1111/jcmm.12923 27515767 PMC5134386

[63] Gomez I Ward B Souilhol C Recarti C Ariaans M Johnston J Neutrophil microvesicles drive atherosclerosis by delivering miR-155 to atheroprone endothelium. Nat Commun. (2020) 11(1):214. 10.1038/s41467-019-14043-y 31924781 PMC6954269

[64] Zhang L Chi J Wu H Xia X Xu C Hao H Extracellular vesicles and endothelial dysfunction in infectious diseases. J Extracell Biol. (2024) 3(4):e148. 10.1002/jex2.148 38938849 PMC11080793

[65] Wang Y Han B Wang Y Wang C Zhang H Xue J Mesenchymal stem cell-secreted extracellular vesicles carrying TGF-β1 up-regulate miR-132 and promote mouse M2 macrophage polarization. J Cell Mol Med. (2020) 24(21):12750–64. 10.1111/jcmm.15860 32965772 PMC7686990

[66] Yang W Yin R Zhu X Yang S Wang J Zhou Z Mesenchymal stem-cell-derived exosomal miR-145 inhibits atherosclerosis by targeting JAM-A. Mol Ther Nucleic Acids. (2021) 23:119–31. 10.1016/j.omtn.2020.10.037 33335797 PMC7732974

[67] Zhang N Luo Y Zhang H Zhang F Gao X Shao J. Exosomes derived from mesenchymal stem cells ameliorate the progression of atherosclerosis in ApoE(−/−) mice via FENDRR. Cardiovasc Toxicol. (2022) 22(6):528–44. 10.1007/s12012-022-09736-8 35344140

[68] Li J Tan M Xiang Q Zhou Z Yan H. Thrombin-activated platelet-derived exosomes regulate endothelial cell expression of ICAM-1 via microRNA-223 during the thrombosis-inflammation response. Thromb Res. (2017) 154:96–105. 10.1016/j.thromres.2017.04.016 28460288

[69] Song T Zhou M Li W Zheng L Wu J Zhao M. Tripeptide leu-ser-trp regulates the vascular endothelial cells phenotype switching by mediating the vascular smooth muscle cells-derived small extracellular vesicles packaging of miR-145. Molecules. (2022) 27(20):7025. 10.3390/molecules27207025 36296612 PMC9610839

[70] Cheng X Zhou H Zhou Y Song C. M2 macrophage-derived exosomes inhibit apoptosis of HUVEC cell through regulating miR-221-3p expression. Biomed Res Int. (2022) 2022:1609244. 10.1155/2022/1609244 36119928 PMC9473890

[71] Jansen F Yang X Baumann K Przybilla D Schmitz T Flender A Endothelial microparticles reduce ICAM-1 expression in a microRNA-222-dependent mechanism. J Cell Mol Med. (2015) 19(9):2202–14. 10.1111/jcmm.12607 26081516 PMC4568925

[72] Zernecke A Bidzhekov K Noels H Shagdarsuren E Gan L Denecke B Delivery of microRNA-126 by apoptotic bodies induces CXCL12-dependent vascular protection. Sci Signal. (2009) 2(100):ra81. 10.1126/scisignal.2000610 19996457

[73] Ke X Liao Z Luo X Chen JQ Deng M Huang Y Endothelial colony-forming cell-derived exosomal miR-21-5p regulates autophagic flux to promote vascular endothelial repair by inhibiting SIPL1A2 in atherosclerosis. Cell Commun Signal. (2022) 20(1):30. 10.1186/s12964-022-00828-0 35279183 PMC8917727

[74] Li J Xue H Li T Chu X Xin D Xiong Y Exosomes derived from mesenchymal stem cells attenuate the progression of atherosclerosis in ApoE(−/−) mice via miR-let7 mediated infiltration and polarization of M2 macrophage. Biochem Biophys Res Commun. (2019) 510(4):565–72. 10.1016/j.bbrc.2019.02.005 30739785

[75] Jiang F Chen Q Wang W Ling Y Yan Y Xia P. Hepatocyte-derived extracellular vesicles promote endothelial inflammation and atherogenesis via microRNA-1. J Hepatol. (2020) 72(1):156–66. 10.1016/j.jhep.2019.09.014 31568800

[76] Quast C Bönner F Polzin A Veulemans V Chennupati R Gyamfi Poku I Aortic valve stenosis causes accumulation of extracellular hemoglobin and systemic endothelial dysfunction. Circulation. (2024) 150(12):952–65. 10.1161/CIRCULATIONAHA.123.064747 38836358

[77] Viola M Bebelman MP Maas RGC de Voogt WS Verweij FJ Seinen CS Hypoxia and TNF-alpha modulate extracellular vesicle release from human induced pluripotent stem cell-derived cardiomyocytes. J Extracell Vesicles. (2024) 13(11):e70000. 10.1002/jev2.70000 39508403 PMC11541862

[78] Cho Y-E Chen S Crouch K Shutt D Kaufman J Singh B. Human breast milk EVs mitigate endothelial dysfunction: preliminary study. *bioRxiv.:2024.05.20.594769* (2024).

[79] Sharma S Mahanty M Rahaman SG Mukherjee P Dutta B Khan MI Avocado-derived extracellular vesicles loaded with ginkgetin and berberine prevent inflammation and macrophage foam cell formation. J Cell Mol Med. (2024) 28(7):e18177. 10.1111/jcmm.18177 38494843 PMC10945093

[80] Cui Z Zhao X Amevor FK Du X Wang Y Li D Therapeutic application of quercetin in aging-related diseases: sIRT1 as a potential mechanism. Front Immunol. (2022) 13:943321. 10.3389/fimmu.2022.943321 35935939 PMC9355713

[81] Sarkar A Mitra S Mehta S Raices R Wewers MD. Monocyte derived microvesicles deliver a cell death message via encapsulated caspase-1. PLoS One. (2009) 4(9):e7140. 10.1371/journal.pone.0007140 19779610 PMC2744928

[82] Tong X Dang X Liu D Wang N Li M Han J Exosome-derived circ_0001785 delays atherogenesis through the ceRNA network mechanism of miR-513a-5p/TGFBR3. J Nanobiotechnol. (2023) 21(1):362. 10.1186/s12951-023-02076-x PMC10548746 37794449

[83] Xu X Lu T Feng Y Cao W Liu D Gao P Carotid plaque-derived small extracellular vesicles mediate atherosclerosis and correlate with plaque vulnerability. MedComm (2020). (2025) 6(6):e70220. 10.1002/mco2.70220 40391195 PMC12086378

[84] Feng W Teng Y Zhong Q Zhang Y Zhang J Zhao P Biomimetic grapefruit-derived extracellular vesicles for safe and targeted delivery of sodium thiosulfate against vascular calcification. ACS Nano. (2023) 17(24):24773–89. 10.1021/acsnano.3c05261 38055864 PMC10753875

[85] Livkisa D Chang TH Burnouf T Czosseck A Le NTN Shamrin G Extracellular vesicles purified from serum-converted human platelet lysates offer strong protection after cardiac ischaemia/reperfusion injury. Biomaterials. (2024) 306:122502. 10.1016/j.biomaterials.2024.122502 38354518

[86] Oggero S de Gaetano M Marcone S Fitzsimons S Pinto AL Ikramova D Extracellular vesicles from monocyte/platelet aggregates modulate human atherosclerotic plaque reactivity. J Extracell Vesicles. (2021) 10(6):12084. 10.1002/jev2.12084 33936566 PMC8077084

[87] Gavriilaki E Lazaridis A Anyfanti P Yiannaki E Dolgyras P Nikolaidou B Circulating microvesicles across a population with various degree of cardiovascular burden are associated with systolic blood pressure. J Hum Hypertens. (2023) 37(12):1105–11. 10.1038/s41371-023-00854-6 37612421

[88] Yu J Tang Y Wang Y Zhou M Li Y Hong J Serum exosomes derived from spontaneously hypertensive rats induce cardiac hypertrophy *in vitro* and *in vivo* by increasing autocrine release of angiotensin II in cardiomyocytes. Biochem Pharmacol. (2023) 210:115462. 10.1016/j.bcp.2023.115462 36849061

[89] Chen X Yan X Gingerich L Chen QH Bi L Shan Z. Induction of neuroinflammation and brain oxidative stress by brain-derived extracellular vesicles from hypertensive rats. Antioxidants (Basel). (2024) 13(3):328. 10.3390/antiox13030328 38539860 PMC10967780

[90] Liu ZZ Jose PA Yang J Zeng C. Importance of extracellular vesicles in hypertension. Exp Biol Med (Maywood). (2021) 246(3):342–53. 10.1177/1535370220974600 33517775 PMC7876642

[91] Zhang C Lu X Hu J Li P Yan J Ling X Bovine milk exosomes alleviate cardiac fibrosis via enhancing angiogenesis *in vivo* and *in vitro*. J Cardiovasc Transl Res. (2022) 15(3):560–70. 10.1007/s12265-021-10174-0 34599486

[92] Ray A Stelloh C Liu Y Meyer A Geurts AM Cowley AW Jr Histone modifications and their contributions to hypertension. Hypertension. (2024) 81(2):229–39. 10.1161/HYPERTENSIONAHA.123.21755 38031837 PMC11229175

[93] Zhang Y Liu Y Liu H Tang WH. Exosomes: biogenesis, biologic function and clinical potential. Cell Biosci. (2019) 9:19. 10.1186/s13578-019-0282-2 30815248 PMC6377728

[94] Perez-Hernandez J Olivares D Forner MJ Ortega A Solaz E Martinez F Urinary exosome miR-146a is a potential marker of albuminuria in essential hypertension. J Transl Med. (2018) 16(1):228. 10.1186/s12967-018-1604-6 30107841 PMC6092786

[95] Lugo-Gavidia LM Carnagarin R Burger D Nolde JM Chan J Robinson S Circulating platelet-derived extracellular vesicles correlate with night-time blood pressure and vascular organ damage and may represent an integrative biomarker of vascular health. J Clin Hypertens (Greenwich). (2022) 24(6):738–49. 10.1111/jch.14479 35502649 PMC9180329

[96] Pironti G Strachan RT Abraham D Mon-Wei Yu S Chen M Chen W Circulating exosomes induced by cardiac pressure overload contain functional angiotensin II type 1 receptors. Circulation. (2015) 131(24):2120–30. 10.1161/CIRCULATIONAHA.115.015687 25995315 PMC4470842

[97] Ren W Liang L Li Y Wei FY Mu N Zhang L Upregulation of miR-423 improves autologous vein graft restenosis via targeting ADAMTS-7. Int J Mol Med. (2020) 45(2):532–42. 10.3892/ijmm.2019.4419 31894258 PMC6984782

[98] Wang C Xing C Li Z Liu Y Li Q Wang Y Bioinspired therapeutic platform based on extracellular vesicles for prevention of arterial wall remodeling in hypertension. Bioact Mater. (2022) 8:494–504. 10.1016/j.bioactmat.2021.06.005 34541415 PMC8427223

[99] Furmanik M van Gorp R Whitehead M Ahmad S Bordoloi J Kapustin A Endoplasmic reticulum stress mediates vascular smooth muscle cell calcification via increased release of Grp78 (glucose-regulated protein, 78 kDa)-loaded extracellular vesicles. Arterioscler Thromb Vasc Biol. (2021) 41(2):898–914. 10.1161/ATVBAHA.120.315506 33297752 PMC7837691

[100] Reel PS Reel S Pearson E Trucco E Jefferson E. Using machine learning approaches for multi-omics data analysis: a review. Biotechnol Adv. (2021) 49:107739. 10.1016/j.biotechadv.2021.107739 33794304

[101] Meor Azlan NF Koeners MP Zhang J. Regulatory control of the Na-Cl co-transporter NCC and its therapeutic potential for hypertension. Acta Pharm Sin B. (2021) 11(5):1117–28. 10.1016/j.apsb.2020.09.009 34094823 PMC8144889

[102] Liao K Yu J Akhmerov A Mohammadigoldar Z Li L Liu W Long noncoding RNA BCYRN1 promotes cardioprotection by enhancing human and murine regulatory T cell dynamics. J Clin Invest. (2025) 135(9):e179262. 10.1172/JCI179262 40131367 PMC12043100

[103] Yuan W Liang X Liu Y Wang H. Mechanism of miR-378a-3p enriched in M2 macrophage-derived extracellular vesicles in cardiomyocyte pyroptosis after MI. Hypertens Res. (2022) 45(4):650–64. 10.1038/s41440-022-00851-1 35082376

[104] Li H Ding J Liu W Wang X Feng Y Guan H Plasma exosomes from patients with acute myocardial infarction alleviate myocardial injury by inhibiting ferroptosis through miR-26b-5p/SLC7A11 axis. Life Sci. (2023) 322:121649. 10.1016/j.lfs.2023.121649 37011873

[105] Wang Y Li C Zhao R Qiu Z Shen C Wang Z Circube3a from M2 macrophage-derived small extracellular vesicles mediates myocardial fibrosis after acute myocardial infarction. Theranostics. (2021) 11(13):6315–33. 10.7150/thno.52843 33995660 PMC8120198

[106] Ge X Meng Q Wei L Liu J Li M Liang X Myocardial ischemia-reperfusion induced cardiac extracellular vesicles harbour proinflammatory features and aggravate heart injury. J Extracell Vesicles. (2021) 10(4):e12072. 10.1002/jev2.12072 33664937 PMC7902529

[107] Wu Y Pan N An Y Xu M Tan L Zhang L. Diagnostic and prognostic biomarkers for myocardial infarction. Front Cardiovasc Med. (2020) 7:617277. 10.3389/fcvm.2020.617277 33614740 PMC7886815

[108] Zhang Z Zou Y Song C Cao K Cai K Chen S Advances in the study of exosomes in cardiovascular diseases. J Adv Res. (2024) 66:133–53. 10.1016/j.jare.2023.12.014 38123019 PMC11674797

[109] Moreno A Alarcón-Zapata P Guzmán-Gútierrez E Radojkovic C Contreras H Nova-Lampeti E Changes in the release of endothelial extracellular vesicles CD144+, CCR6+, and CXCR3+ in individuals with acute myocardial infarction. Biomedicines. (2024) 12(9):2119. 10.3390/biomedicines12092119 39335632 PMC11430588

[110] Anselmo A Frank D Papa L Viviani Anselmi C Di Pasquale E Mazzola M Myocardial hypoxic stress mediates functional cardiac extracellular vesicle release. Eur Heart J. (2021) 42(28):2780–92. 10.1093/eurheartj/ehab247 34104945

[111] Ondracek AS Afonyushkin T Aszlan A Taqi S Koller T Artner T Malondialdehyde-specific natural IgM inhibit NETosis triggered by culprit site-derived extracellular vesicles from myocardial infarction patients. Eur Heart J. (2025) 46(10):926–39. 10.1093/eurheartj/ehae584 39215577 PMC11887544

[112] Jin X Xu W Wu Q Huang C Song Y Lian J. Detecting early-warning biomarkers associated with heart-exosome genetic-signature for acute myocardial infarction: a source-tracking study of exosome. J Cell Mol Med. (2024) 28(8):e18334. 10.1111/jcmm.18334 38661439 PMC11044819

[113] Bouchareychas L Duong P Phu TA Alsop E Meechoovet B Reiman R High glucose macrophage exosomes enhance atherosclerosis by driving cellular proliferation & hematopoiesis. iScience. (2021) 24(8):102847. 10.1016/j.isci.2021.102847 34381972 PMC8333149

[114] Sharma P Roy A Dhamija RK Bhushan S Baswal K Kulandaisamy R A comprehensive proteomic profiling of urinary exosomes and the identification of early non-invasive biomarker in patients with coronary artery disease. J Proteomics. (2024) 293:105059. 10.1016/j.jprot.2023.105059 38151158

[115] Whiteside TL. Proteomic analysis of plasma-derived exosomes in defining their role as biomarkers of disease progression, response to therapy and outcome. Proteomes. (2019) 7(3):27. 10.3390/proteomes7030027 31262017 PMC6789797

[116] Zhang Q Ren T Cao K Xu Z. Advances of machine learning-assisted small extracellular vesicles detection strategy. Biosens Bioelectron. (2024) 251:116076. 10.1016/j.bios.2024.116076 38340580

[117] Kenneweg F Bang C Xiao K Boulanger CM Loyer X Mazlan S Long noncoding RNA-enriched vesicles secreted by hypoxic cardiomyocytes drive cardiac fibrosis. Mol Ther Nucleic Acids. (2019) 18:363–74. 10.1016/j.omtn.2019.09.003 31634682 PMC6807307

[118] Yang Y Cai Y Wu G Chen X Liu Y Wang X Plasma long non-coding RNA, CoroMarker, a novel biomarker for diagnosis of coronary artery disease. Clin Sci (Lond). (2015) 129(8):675–85. 10.1042/CS20150121 26201019

[119] Jansen F Yang X Proebsting S Hoelscher M Przybilla D Baumann K MicroRNA expression in circulating microvesicles predicts cardiovascular events in patients with coronary artery disease. J Am Heart Assoc. (2014) 3(6):e001249. 10.1161/JAHA.114.001249 25349183 PMC4338711

[120] Fitzsimons S Oggero S Bruen R McCarthy C Strowitzki MJ Mahon NG microRNA-155 is decreased during atherosclerosis regression and is increased in urinary extracellular vesicles during atherosclerosis progression. Front Immunol. (2020) 11:576516. 10.3389/fimmu.2020.576516 33391256 PMC7773661

[121] Liu Y Li Q Hosen MR Zietzer A Flender A Levermann P Atherosclerotic conditions promote the packaging of functional MicroRNA-92a-3p into endothelial microvesicles. Circ Res. (2019) 124(4):575–87. 10.1161/CIRCRESAHA.118.314010 30582459

[122] D'Alessandra Y Devanna P Limana F Straino S Carlo D Brambilla A Circulating microRNAs are new and sensitive biomarkers of myocardial infarction. Eur Heart J. (2010) 31(22):2765–73. 10.1093/eurheartj/ehq167 20534597 PMC2980809

[123] Matsumoto S Sakata Y Suna S Nakatani D Usami M Hara M Circulating p53-responsive microRNAs are predictive indicators of heart failure after acute myocardial infarction. Circ Res. (2013) 113(3):322–6. 10.1161/CIRCRESAHA.113.301209 23743335

[124] Emanueli C Shearn AI Laftah A Fiorentino F Reeves BC Beltrami C Coronary artery-bypass-graft surgery increases the plasma concentration of exosomes carrying a cargo of cardiac MicroRNAs: an example of exosome trafficking out of the human heart with potential for cardiac biomarker discovery. PLoS One. (2016) 11(4):e0154274. 10.1371/journal.pone.0154274 27128471 PMC4851293

[125] Ji Q Ji Y Peng J Zhou X Chen X Zhao H Increased brain-specific MiR-9 and MiR-124 in the serum exosomes of acute ischemic stroke patients. PLoS One. (2016) 11(9):e0163645. 10.1371/journal.pone.0163645 27661079 PMC5035015

[126] Zhou J Chen L Chen B Huang S Zeng C Wu H Increased serum exosomal miR-134 expression in the acute ischemic stroke patients. BMC Neurol. (2018) 18(1):198. 10.1186/s12883-018-1196-z 30514242 PMC6278025

[127] Song P Sun H Chen H Wang Y Zhang Q. Decreased serum exosomal miR-152-3p contributes to the progression of acute ischemic stroke. Clin Lab. (2020) 66(8). 10.7754/Clin.Lab.2020.200106 32776748

[128] Wang W Li DB Li RY Zhou X Yu DJ Lan XY Diagnosis of hyperacute and acute ischaemic stroke: the potential utility of exosomal MicroRNA-21-5p and MicroRNA-30a-5p. Cerebrovasc Dis. (2018) 45(5-6):204–12. 10.1159/000488365 29627835

[129] Li DB Liu JL Wang W Li RY Yu DJ Lan XY Plasma exosomal miR-422a and miR-125b-2-3p serve as biomarkers for ischemic stroke. Curr Neurovasc Res. (2017) 14(4):330–7. 10.2174/1567202614666171005153434 28982331

[130] Chen Y Song Y Huang J Qu M Zhang Y Geng J Increased circulating exosomal miRNA-223 is associated with acute ischemic stroke. Front Neurol. (2017) 8:57. 10.3389/fneur.2017.00057 28289400 PMC5326773

[131] Pir GJ Zahid MA Akhtar N Ayadathil R Pananchikkal SV Joseph S Differentially expressed miRNA profiles of serum derived extracellular vesicles from patients with acute ischemic stroke. Brain Res. (2024) 1845:149171. 10.1016/j.brainres.2024.149171 39168264

[132] Sinning JM Losch J Walenta K Böhm M Nickenig G Werner N. Circulating CD31+/annexin V+ microparticles correlate with cardiovascular outcomes. Eur Heart J. (2011) 32(16):2034–41. 10.1093/eurheartj/ehq478 21186238

[133] Cheow ES Cheng WC Lee CN de Kleijn D Sorokin V Sze SK. Plasma-derived extracellular vesicles contain predictive biomarkers and potential therapeutic targets for myocardial ischemic (MI) injury. Mol Cell Proteomics. (2016) 15(8):2628–40. 10.1074/mcp.M115.055731 27234505 PMC4974341

[134] Sun X Jung JH Arvola O Santoso MR Giffard RG Yang PC Stem cell-derived exosomes protect astrocyte cultures from *in vitro* ischemia and decrease injury as post-stroke intravenous therapy. Front Cell Neurosci. (2019) 13:394. 10.3389/fncel.2019.00394 31551712 PMC6733914

[135] Liu L Jin X Hu CF Li R Zhou Z Shen CX. Exosomes derived from mesenchymal stem cells rescue myocardial ischaemia/reperfusion injury by inducing cardiomyocyte autophagy via AMPK and akt pathways. Cell Physiol Biochem. (2017) 43(1):52–68. 10.1159/000480317 28848091

[136] Sun L Ji Y Chi B Xiao T Li C Yan X A 3D culture system improves the yield of MSCs-derived extracellular vesicles and enhances their therapeutic efficacy for heart repair. Biomed Pharmacother. (2023) 161:114557. 10.1016/j.biopha.2023.114557 36963364

[137] Zhang N Song Y Huang Z Chen J Tan H Yang H Monocyte mimics improve mesenchymal stem cell-derived extracellular vesicle homing in a mouse MI/RI model. Biomaterials. (2020) 255:120168. 10.1016/j.biomaterials.2020.120168 32562944

[138] Zhu D Wang Y Thomas M McLaughlin K Oguljahan B Henderson J Exosomes from adipose-derived stem cells alleviate myocardial infarction via microRNA-31/FIH1/HIF-1α pathway. J Mol Cell Cardiol. (2022) 162:10–9. 10.1016/j.yjmcc.2021.08.010 34474073 PMC8766956

[139] Wang T Li T Niu X Hu L Cheng J Guo D ADSC-derived exosomes attenuate myocardial infarction injury by promoting miR-205-mediated cardiac angiogenesis. Biol Direct. (2023) 18(1):6. 10.1186/s13062-023-00361-1 36849959 PMC9972746

[140] de Almeida Oliveira NC Neri EA Silva CM Valadão IC Fonseca-Alaniz MH Zogbi C Multicellular regulation of miR-196a-5p and miR-425-5 from adipose stem cell-derived exosomes and cardiac repair. Clin Sci (Lond). (2022) 136(17):1281–301. 10.1042/CS20220216 35894060

[141] Zhang J Zhang J Jiang X Jin J Wang H Zhang Q. ASCs-EVs inhibit apoptosis and promote myocardial function in the infarcted heart via miR-221. Discov Med. (2023) 35(179):1077–85. 10.24976/Discov.Med.202335179.104 38058073

[142] Ciullo A Li L Li C Tsi K Farrell C Pellegrini M Non-coding RNA yREX3 from human extracellular vesicles exerts macrophage-mediated cardioprotection via a novel gene-methylating mechanism. Eur Heart J. (2024) 45(29):2660–73. 10.1093/eurheartj/ehae357 38865332 PMC11297535

[143] Li H Liu Y Lin Y Li S Liu C Cai A Cardiac repair using regenerating neonatal heart tissue-derived extracellular vesicles. Nat Commun. (2025) 16(1):1292. 10.1038/s41467-025-56384-x 39900896 PMC11790877

[144] Costa A Balbi C Garbati P Palamà MEF Reverberi D De Palma A Investigating the paracrine role of perinatal derivatives: human amniotic fluid stem cell-extracellular vesicles show promising transient potential for cardiomyocyte renewal. Front Bioeng Biotechnol. (2022) 10:902038. 10.3389/fbioe.2022.902038 35757808 PMC9214211

[145] Wang N Chen C Yang D Liao Q Luo H Wang X Mesenchymal stem cells-derived extracellular vesicles, via miR-210, improve infarcted cardiac function by promotion of angiogenesis. Biochim Biophys Acta Mol Basis Dis. (2017) 1863(8):2085–92. 10.1016/j.bbadis.2017.02.023 28249798

[146] Ning H Chen H Deng J Xiao C Xu M Shan L Exosomes secreted by FNDC5-BMMSCs protect myocardial infarction by anti-inflammation and macrophage polarization via NF-κB signaling pathway and Nrf2/HO-1 axis. Stem Cell Res Ther. (2021) 12(1):519. 10.1186/s13287-021-02591-4 34583757 PMC8480009

[147] Yang M Liao M Liu R Zhang Q Zhang S He Y Human umbilical cord mesenchymal stem cell-derived extracellular vesicles loaded with miR-223 ameliorate myocardial infarction through P53/S100A9 axis. Genomics. (2022) 114(3):110319. 10.1016/j.ygeno.2022.110319 35227836

[148] Wu Y Peng W Fang M Wu M Wu M. MSCs-derived extracellular vesicles carrying miR-212-5p alleviate myocardial infarction-induced cardiac fibrosis via NLRC5/VEGF/TGF-β1/SMAD axis. J Cardiovasc Transl Res. (2022) 15(2):302–16. 10.1007/s12265-021-10156-2 34508321

[149] Huang C Chng WH Neupane YR Lai Y Cui W Yang M Adipose stem cell-derived nanovesicles for cardioprotection: production and identification of therapeutic components. J Control Release. (2025) 385:113989. 10.1016/j.jconrel.2025.113989 40582645

[150] Mao CY Zhang TT Li DJ Zhou E Fan YQ He Q Extracellular vesicles from hypoxia-preconditioned mesenchymal stem cells alleviates myocardial injury by targeting thioredoxin-interacting protein-mediated hypoxia-inducible factor-1α pathway. World J Stem Cells. (2022) 14(2):183–99. 10.4252/wjsc.v14.i2.183 35432732 PMC8963381

[151] Jang E Yu H Kim E Hwang J Yoo J Choi J The therapeutic effects of blueberry-treated stem cell-derived extracellular vesicles in ischemic stroke. Int J Mol Sci. (2024) 25(12):6362. 10.3390/ijms25126362 38928069 PMC11203670

[152] Chen S Zeng X Wu M Zhu J Wu Y. Sodium alginate hydrogel infusion of bone marrow mesenchymal stem cell-derived extracellular vesicles and p38α antagonistic peptides in myocardial infarction fibrosis mitigation. J Am Heart Assoc. (2025) 14(8):e036887. 10.1161/JAHA.124.036887 40178108 PMC12184616

[153] Pu Y Li C Qi X Xu R Dong L Jiang Y Extracellular vesicles from NMN preconditioned mesenchymal stem cells ameliorated myocardial infarction via miR-210-3p promoted angiogenesis. Stem Cell Rev Rep. (2023) 19(4):1051–66. 10.1007/s12015-022-10499-6 36696015 PMC10185590

[154] Zhang Y Yang N Huang X Zhu Y Gao S Liu Z Melatonin engineered adipose-derived biomimetic nanovesicles regulate mitochondrial functions and promote myocardial repair in myocardial infarction. Front Cardiovasc Med. (2022) 9:789203. 10.3389/fcvm.2022.789203 35402545 PMC8985816

[155] Zhang C Wang H Chan GCF Zhou Y Lai X Lian M. Extracellular vesicles derived from human umbilical cord mesenchymal stromal cells protect cardiac cells against hypoxia/reoxygenation injury by inhibiting endoplasmic Reticulum stress via activation of the PI3K/akt pathway. Cell Transplant. (2020) 29:963689720945677. 10.1177/0963689720945677 32864999 PMC7563023

[156] Adamiak M Cheng G Bobis-Wozowicz S Zhao L Kedracka-Krok S Samanta A Induced pluripotent stem cell (iPSC)-derived extracellular vesicles are safer and more effective for cardiac repair than iPSCs. Circ Res. (2018) 122(2):296–309. 10.1161/CIRCRESAHA.117.311769 29118058 PMC5775034

[157] Jung JH Ikeda G Tada Y von Bornstädt D Santoso MR Wahlquist C miR-106a-363 cluster in extracellular vesicles promotes endogenous myocardial repair via Notch3 pathway in ischemic heart injury. Basic Res Cardiol. (2021) 116(1):19. 10.1007/s00395-021-00858-8 33742276 PMC8601755

[158] Kesidou D Bennett M Monteiro JP McCracken IR Klimi E Rodor J Extracellular vesicles from differentiated stem cells contain novel proangiogenic miRNAs and induce angiogenic responses at low doses. Mol Ther. (2024) 32(1):185–203. 10.1016/j.ymthe.2023.11.023 38096818 PMC10787168

[159] Wei Z Chen Z Zhao Y Fan F Xiong W Song S Mononuclear phagocyte system blockade using extracellular vesicles modified with CD47 on membrane surface for myocardial infarction reperfusion injury treatment. Biomaterials. (2021) 275:121000. 10.1016/j.biomaterials.2021.121000 34218049

[160] Kang JY Kim H Mun D Yun N Joung B. Co-delivery of curcumin and miRNA-144-3p using heart-targeted extracellular vesicles enhances the therapeutic efficacy for myocardial infarction. J Control Release. (2021) 331:62–73. 10.1016/j.jconrel.2021.01.018 33460670

[161] Wang Y Meng D Shi X Hou Y Zang S Chen L Injectable hydrogel with miR-222-engineered extracellular vesicles ameliorates myocardial ischemic reperfusion injury via mechanotransduction. Cell Rep Med. (2025) 6(3):101987. 10.1016/j.xcrm.2025.101987 40037358 PMC11970392

[162] Tian C Gao L Rudebush TL Yu L Zucker IH. Extracellular vesicles regulate sympatho-excitation by Nrf2 in heart failure. Circ Res. (2022) 131(8):687–700. 10.1161/CIRCRESAHA.122.320916 36098045 PMC9529870

[163] Senesi G Lodrini AM Mohammed S Mosole S Hjortnaes J Veltrop RJA miR-24-3p secreted as extracellular vesicle cargo by cardiomyocytes inhibits fibrosis in human cardiac microtissues. Cardiovasc Res. (2025) 121(1):143–56. 10.1093/cvr/cvae243 39527589 PMC11998913

[164] Zhu D Liu S Huang K Li J Mei X Li Z Intrapericardial long non-coding RNA-Tcf21 antisense RNA inducing demethylation administration promotes cardiac repair. Eur Heart J. (2023) 44(19):1748–60. 10.1093/eurheartj/ehad114 36916305 PMC10411945

[165] Wei G Li C Jia X Xie J Tang Z Jin M Extracellular vesicle-derived CircWhsc1 promotes cardiomyocyte proliferation and heart repair by activating TRIM59/STAT3/cyclin B2 pathway. J Adv Res. (2023) 53:199–218. 10.1016/j.jare.2022.12.014 36587763 PMC10658329

[166] Lim XC Huang C Yatim S Chong SY Tan SH Yang X Temporal changes in extracellular vesicle hemostatic protein composition predict favourable left ventricular remodeling after acute myocardial infarction. Int J Mol Sci. (2022) 24(1):327. 10.3390/ijms24010327 36613770 PMC9820565

[167] Yu L Liang Y Zhang M Yang PC Hinek A Mao S. Extracellular vesicle-derived circCEBPZOS attenuates postmyocardial infarction remodeling by promoting angiogenesis via the miR-1178-3p/PDPK1 axis. Commun Biol. (2023) 6(1):133. 10.1038/s42003-023-04505-x 36726025 PMC9892031

[168] Kim H Kim DE Han G Lim NR Kim EH Jang Y Harnessing the natural healing power of colostrum: bovine milk-derived extracellular vesicles from colostrum facilitating the transition from inflammation to tissue regeneration for accelerating cutaneous wound healing. Adv Healthc Mater. (2022) 11(6):e2102027. 10.1002/adhm.202102027 34865307 PMC11468066

[169] Min XL Jia WJ Guo L Jing R Zhao XH Hu JY Brain microvascular endothelial cell-derived exosomes transmitting circ_0000495 promote microglial M1-polarization and endothelial cell injury under hypoxia condition. FASEB J. (2024) 38(2):e23387. 10.1096/fj.202301637R 38193649

[170] Zhang Y Liu Z Chopp M Millman M Li Y Cepparulo P Small extracellular vesicles derived from cerebral endothelial cells with elevated microRNA 27a promote ischemic stroke recovery. Neural Regen Res. (2025) 20(1):224–33. 10.4103/NRR.NRR-D-22-01292 38767487 PMC11246145

[171] Xin D Li T Zhao Y Guo X Gai C Jiang Z MiR-100-5p-rich small extracellular vesicles from activated neuron to aggravate microglial activation and neuronal activity after stroke. J Nanobiotechnol. (2024) 22(1):534. 10.1186/s12951-024-02782-0 PMC11370036 39227960

[172] Li Z Song Y He T Wen R Li Y Chen T M2 microglial small extracellular vesicles reduce glial scar formation via the miR-124/STAT3 pathway after ischemic stroke in mice. Theranostics. (2021) 11(3):1232–48. 10.7150/thno.48761 33391532 PMC7738903

[173] Zhang Q Yi Y Chen T Ai Y Chen Z Liu G M2 microglia-derived small extracellular vesicles modulate NSC fate after ischemic stroke via miR-25-3p/miR-93-5p-TGFBR/PTEN/FOXO3 axis. J Nanobiotechnol. (2025) 23(1):311. 10.1186/s12951-025-03390-2 PMC12020034 40270025

[174] Hira K Ueno Y Tanaka R Miyamoto N Yamashiro K Inaba T Astrocyte-derived exosomes treated with a semaphorin 3A inhibitor enhance stroke recovery via prostaglandin D(2) synthase. Stroke. (2018) 49(10):2483–94. 10.1161/STROKEAHA.118.021272 30355116

[175] Rehni AK Cho S Quero HN Shukla V Zhang Z Dong C Red blood cell microparticles limit hematoma growth in intracerebral hemorrhage. Stroke. (2022) 53(10):3182–91. 10.1161/STROKEAHA.122.039641 36069183 PMC9529820

[176] Edwardson MA Mitsuhashi M Van Epps D. Elevation of astrocyte-derived extracellular vesicles over the first month post-stroke in humans. Sci Rep. (2024) 14(1):5272. 10.1038/s41598-024-55983-w 38438491 PMC10912590

[177] Li Y Liu B Zhao T Quan X Han Y Cheng Y Comparative study of extracellular vesicles derived from mesenchymal stem cells and brain endothelial cells attenuating blood-brain barrier permeability via regulating Caveolin-1-dependent ZO-1 and Claudin-5 endocytosis in acute ischemic stroke. J Nanobiotechnol. (2023) 21(1):70. 10.1186/s12951-023-01828-z PMC9976550 36855156

[178] Wang Y Niu H Li L Han J Liu Z Chu M Anti-CHAC1 exosomes for nose-to-brain delivery of miR-760-3p in cerebral ischemia/reperfusion injury mice inhibiting neuron ferroptosis. J Nanobiotechnol. (2023) 21(1):109. 10.1186/s12951-023-01862-x PMC10041751 36967397

[179] Miao Y Liang X Chen J Liu H He Z Qin Y Transfer of miR-877-3p via extracellular vesicles derived from dental pulp stem cells attenuates neuronal apoptosis and facilitates early neurological functional recovery after cerebral ischemia-reperfusion injury through the Bclaf1/P53 signaling pathway. Pharmacol Res. (2024) 206:107266. 10.1016/j.phrs.2024.107266 38878918

[180] Li S Zhang R Wang A Li Y Zhang M Kim J Panax notoginseng: derived exosome-like nanoparticles attenuate ischemia reperfusion injury via altering microglia polarization. J Nanobiotechnol. (2023) 21(1):416. 10.1186/s12951-023-02161-1 PMC10636993 37946257

[181] Cai H Huang LY Hong R Song JX Guo XJ Zhou W Momordica charantia exosome-like nanoparticles exert neuroprotective effects against ischemic brain injury via inhibiting matrix metalloproteinase 9 and activating the AKT/GSK3β signaling pathway. Front Pharmacol. (2022) 13:908830. 10.3389/fphar.2022.908830 35814200 PMC9263912

[182] Luo H Ye G Liu Y Huang D Luo Q Chen W miR-150-3p enhances neuroprotective effects of neural stem cell exosomes after hypoxic-ischemic brain injury by targeting CASP2. Neurosci Lett. (2022) 779:136635. 10.1016/j.neulet.2022.136635 35436510

[183] Pan J Wang Z Huang X Xue J Zhang S Guo X Bacteria-derived outer-membrane vesicles hitchhike neutrophils to enhance ischemic stroke therapy. Adv Mater. (2023) 35(38):e2301779. 10.1002/adma.202301779 37358255

[184] Yang Z Gao Z Yang Z Zhang Y Chen H Yang X Lactobacillus plantarum-derived extracellular vesicles protect against ischemic brain injury via the microRNA-101a-3p/c-Fos/TGF-β axis. Pharmacol Res. (2022) 182:106332. 10.1016/j.phrs.2022.106332 35779817

[185] Shi X Zhang L Wu S Zhang C Mamtilahun M Li Y A simple polydopamine-based platform for engineering extracellular vesicles with brain-targeting peptide and imaging probes to improve stroke outcome. J Extracell Vesicles. (2025) 14(1):e70031. 10.1002/jev2.70031 39783851 PMC11714163

[186] Qin Z Liao R Xiong Y Jiang L Li J Wang L A narrative review of exosomes in vascular calcification. Ann Transl Med. (2021) 9(7):579. 10.21037/atm-20-7355 33987277 PMC8105793

[187] Xu D Di K Fan B Wu J Gu X Sun Y MicroRNAs in extracellular vesicles: sorting mechanisms, diagnostic value, isolation, and detection technology. Front Bioeng Biotechnol. (2022) 10:948959. 10.3389/fbioe.2022.948959 36324901 PMC9618890

[188] Zhao W Zhao SP Zhao YH. MicroRNA-143/-145 in cardiovascular diseases. Biomed Res Int. (2015) 2015:531740. 10.1155/2015/531740 26221598 PMC4499377

[189] Piccoli M Cirillo F Ghiroldi A Rota P Coviello S Tarantino A Sphingolipids and atherosclerosis: the dual role of ceramide and sphingosine-1-phosphate. Antioxidants (Basel). (2023) 12(1):143. 10.3390/antiox12010143 36671005 PMC9855164

[190] Hmadeh S Trimaille A Matsushita K Marchandot B Carmona A Zobairi F Human aortic stenotic valve-derived extracellular vesicles induce endothelial dysfunction and thrombogenicity through AT1R/NADPH oxidases/SGLT2 pro-oxidant pathway. JACC Basic Transl Sci. (2024) 9(7):845–64. 10.1016/j.jacbts.2024.02.012 39170957 PMC11334416

[191] Beaumier A Robinson SR Robinson N Lopez KE Meola DM Barber LG Extracellular vesicular microRNAs as potential biomarker for early detection of doxorubicin-induced cardiotoxicity. J Vet Intern Med. (2020) 34(3):1260–71. 10.1111/jvim.15762 32255536 PMC7255649

[192] Gupta M Tieu A Slobodian M Shorr R Burger D Lalu MM Preclinical studies of MSC-derived extracellular vesicles to treat or prevent graft versus host disease: a systematic review of the literature. Stem Cell Rev Rep. (2021) 17(2):332–40. 10.1007/s12015-020-10058-x 33159616 PMC7648545

[193] Dutta P Lincoln J. Calcific aortic valve disease: a developmental biology perspective. Curr Cardiol Rep. (2018) 20(4):21. 10.1007/s11886-018-0968-9 29520694 PMC5842494

[194] Yang R Tang Y Chen X Yang Y. Telocytes-derived extracellular vesicles alleviate aortic valve calcification by carrying miR-30b. ESC Heart Fail. (2021) 8(5):3935–46. 10.1002/ehf2.13460 34165260 PMC8497371

[195] Wight TN Potter-Perigo S. The extracellular matrix: an active or passive player in fibrosis? Am J Physiol Gastrointest Liver Physiol. (2011) 301(6):G950–5. 10.1152/ajpgi.00132.2011 21512158 PMC3233785

[196] Hwang HS Kim H Han G Lee JW Kim K Kwon IC Extracellular vesicles as potential therapeutics for inflammatory diseases. Int J Mol Sci. (2021) 22(11):5487. 10.3390/ijms22115487 34067503 PMC8196952

[197] Meng W He C Hao Y Wang L Li L Zhu G. Prospects and challenges of extracellular vesicle-based drug delivery system: considering cell source. Drug Deliv. (2020) 27(1):585–98. 10.1080/10717544.2020.1748758 32264719 PMC7178886

[198] Su X Wang H Li Q Chen Z. Extracellular vesicles: a review of their therapeutic potentials, sources, biodistribution, and administration routes. Int J Nanomed. (2025) 20:3175–99. 10.2147/IJN.S502591 PMC11913029 40098717

[199] Kang M Jordan V Blenkiron C Chamley LW. Biodistribution of extracellular vesicles following administration into animals: a systematic review. J Extracell Vesicles. (2021) 10(8):e12085. 10.1002/jev2.12085 34194679 PMC8224174

[200] Cappucci IP Tremoli E Zavan B Ferroni L. Circulating extracellular vesicles in cardiovascular disease. Int J Mol Sci. (2025) 26(14):6817. 10.3390/ijms26146817 40725065 PMC12295213

[201] Witwer KW Goberdhan DC O'Driscoll L Théry C Welsh JA Blenkiron C Updating MISEV: evolving the minimal requirements for studies of extracellular vesicles. J Extracell Vesicles. (2021) 10(14):e12182. 10.1002/jev2.12182 34953156 PMC8710080

[202] Chitoiu L Dobranici A Gherghiceanu M Dinescu S Costache M. Multi-omics data integration in extracellular vesicle biology-utopia or future reality? Int J Mol Sci. (2020) 21(22):8550. 10.3390/ijms21228550 33202771 PMC7697477

[203] Khan AA Rosales T. Radiolabelling of extracellular vesicles for PET and SPECT imaging. Nanotheranostics. (2021) 5(3):256–74. 10.7150/ntno.51676 33654653 PMC7914338

